# Identification and validation of platelet-related diagnostic markers and potential drug screening in ischemic stroke by integrating comprehensive bioinformatics analysis and machine learning

**DOI:** 10.3389/fimmu.2023.1320475

**Published:** 2024-01-10

**Authors:** Yifei Geng, Yuchen Liu, Min Wang, Xi Dong, Xiao Sun, Yun Luo, Xiaobo Sun

**Affiliations:** ^1^ Institute of Medicinal Plant Development, Peking Union Medical College and Chinese Academy of Medical Sciences, Beijing, China; ^2^ Key Laboratory of Bioactive Substances and Resources Utilization of Chinese Herbal Medicine, Ministry of Education, Beijing, China; ^3^ Institute of Medicinal Plant Development, Peking Union Medical College and Chinese Academy of Medical Sciences, Beijing Key Laboratory of Innovative Drug Discovery of Traditional Chinese Medicine (Natural Medicine) and Translational Medicine, Beijing, China; ^4^ Department of Internal Medicine, Peking Union Medical College Hospital, Beijing, China; ^5^ School of Clinical Science, Peking Union Medical College, Chinese Academy of Medical Science, Beijing, China

**Keywords:** stroke, platelet, bioinformatics, machine learning, drug screening

## Abstract

**Background:**

Ischemic stroke (IS), caused by blood and oxygen deprivation due to cerebral thrombosis, has links to activated and aggregated platelets. Discovering platelet-related biomarkers, developing diagnostic models, and screening antiplatelet drugs are crucial for IS diagnosis and treatment.

**Methods and results:**

Combining and normalizing GSE16561 and GSE22255 datasets identified 1,753 upregulated and 1,187 downregulated genes. Fifty-one genes in the platelet-related module were isolated using weighted gene co-expression network analysis (WGCNA) and other analyses, including 50 upregulated and one downregulated gene. Subsequent enrichment and network analyses resulted in 25 platelet-associated genes and six diagnostic markers for a risk assessment model. This model’s area under the ROC curve outperformed single genes, and in the peripheral blood of the high-risk group, immune infiltration indicated a higher proportion of CD4, resting CD4 memory, and activated CD4 memory T cells, along with a lower proportion of CD8 T cells in comparison to the low-risk group. Utilizing the gene expression matrix and the CMap database, we identified two potential drugs for IS. Finally, a rat MACO/R model was used to validate the diagnostic markers’ expression and the drugs’ predicted anticoagulant effects.

**Conclusion:**

We identified six IS platelet-related biomarkers (APP, THBS1, F13A1, SRC, PPBP, and VCL) for a robust diagnostic model. The drugs alpha-linolenic acid and ciprofibrate have potential antiplatelet effects in IS. This study advances early IS diagnosis and treatment.

## Introduction

Stroke, the global second most prominent cause of death following ischemic heart disease, is an abrupt onslaught on the nervous system. Etiologically, it is traceable to cerebral vascular reperfusion damage, a subset of cerebrovascular ailments (1–3). Ischemic stroke (IS), characterized primarily by cerebral vascular thrombosis, precipitates an inadequate distribution of blood and oxygen to the brain (4). IS screening and diagnosis are conducted by employing facial, arm, and speech tests (FAST) and various advanced medical imaging techniques. Nevertheless, the potential for diagnostic failure or deferred imaging examination is introduced by factors such as atypical symptomatology in stroke patients, physical discomfort, emotional distress, and the limited sensitivity of diagnostic equipment (5).

In the therapeutic landscape, recombinant tissue plasminogen activator (tPA) thrombolysis is the sole FDA-endorsed IS treatment (6). Clinical outcomes show significant improvement when 0.9 mg/kg of alteplase is administered intravenously within a 4.5-h stroke onset (5). Concurrently, calcium antagonists administered intravenously and orally active antihypertensives regulate patients’ blood pressure, while active bleeding in patients with acute hemorrhagic stroke is meticulously managed. Severe cases may incline toward surgical interventions, such as craniotomy and neuroendoscopic surgeries (7). Despite these advancements, the efficacy of tPA therapy is hampered by the narrow time window within which the treatment can be administered, a situation further compounded by patient-related factors such as delayed hospital arrival (8). Furthermore, surgical intervention has not substantially improved mortality rates or patient prognosis (9).

Within the sphere of thrombotic disorders, platelets, being infinitesimal cell fragments, are cardinal actors in thrombus generation and are the core focus of antithrombotic therapy (10). Recent research has elucidated that platelets are activated through various agonists, such as reactive oxygen species, von Willebrand factor, and damage-associated molecular pattern molecules, in the wake of ischemia/reperfusion (I/R) damage (11). Preliminary stages of cerebral vessel thrombus formation involve the accumulation of platelet-bound red blood cells and certain coagulation factors at the injury site, thereby giving rise to a developing, porous, protein-scaffolded thrombus. However, subsequent accruement of platelets and fibrin metamorphoses the thrombus into a dense, stable, and high-occlusion structure. Such development amplifies the resistance of the thrombus to thrombolysis and diminishes tPA penetration, thereby presenting a significant clinical challenge to IS treatment (12). While antiplatelet agents (like aspirin and clopidogrel) are enlisted as secondary prevention treatments for stroke, alternative antithrombotic therapies not targeting platelets have comprehensively demonstrated efficacy only for atrial fibrillation (11). Consequently, the link between cerebral ischemic damage and platelets is gaining extensive scientific focus in the quest for viable diagnostic and therapeutic targets.

Over recent decades, the advent of multi-omics, sundry artificial intelligence mechanisms, and data-driven technologies has extensively propelled the discovery of medical diagnostic and prognostic markers and the screening of prospective drug candidates (13). Based on the intricate and high-dimensional nature of datasets related to cerebral ischemia, the imperative of employing machine learning—a suite of mathematical approaches devised to extract knowledge and insights from expansive datasets—has become more apparent (13) Computer-Assisted Drug Design (CADD), which utilizes computational technology and software to enhance the identification of potential drug candidates backed by the structural understanding of target molecules (structure-based) or established ligands with biological functions (ligand-based), is becoming fundamental (14). In recent years, there has been significant exploration of expression data pertaining to IS. Zhang et al. conducted a comprehensive analysis, determining that the differentially expressed genes within the IS patient dataset were notably enriched in two pathways, namely, oxidative phosphorylation and Alzheimer’s disease (15). Furthermore, the validation of key genes was performed using quantitative real-time polymerase chain reaction (qRT-PCR) (15). Additionally, Yang et al. employed a weighted co-expression network analysis to categorize patients with acute IS into three subgroups. This classification facilitates tailored treatment based on their peripheral blood immune status (16). Several studies have utilized weighted gene co-expression network analysis (WGCNA) to identify immune-related genes and cell death-related biomarkers, which play pivotal roles in the progression of IS (17, 18). Concurrently, the networks associated with ciRNA, miRNA-mRNA, and neutrophils have been established, shedding light on the pertinent gene biomarkers (19, 20). Despite the previous bioinformatics analyses reporting various cell death modes and immunophenotypes in IS, a systematic analysis of platelets, integral targets in the coagulation and antithrombotic processes of IS, and their related drugs, remains outstanding. This endeavor, processed through a meshwork of bioinformatics and machine learning, aims to establish a validated approach to drug screening premised on the genes associated with this model.

## Materials and methods

### Data sources and processing

The GSE16561 and GSE22255 datasets were retrieved from the Gene Expression Omnibus database (http://www.ncbi.nlm.nih.gov/geo/) (21). These cohorts include peripheral blood samples from IS patients. Comprehensive details regarding these datasets are briefly summarized in **Table 1**. The inclusion criteria for the dataset in this study comprised the following: (a) the dataset needed to encompass a genome-wide mRNA expression profile. (b) The data source should originate from peripheral blood samples obtained from individuals within the IS group and samples from healthy population controls that had not been stimulated with drugs and transfected. (c) The dataset had to be original and complete. (d) The dataset type was specifically designated as “expression profiling by array.”

**Table 1:** Basic information about the datasets.

Datasets	Platform	IS samples	Control samples	Sequencing type	Source
GSE16561	GPL6883	39	24	mRNA	Peripheral blood
GSE22255	GPL570	20	20	mRNA	Peripheral blood

Moreover, the identifier conversion for expression profiling arrays of GSE22255 and GSE16561 was achieved employing the “hgu133plus2.db” Platform annotation file, alongside the “tidyverse” and “AnnoProbe” packages sourced from R. After their generation, the expression profiles were consolidated utilizing the Combat function derived from the “sva” package to neutralize potential batch discrepancies. An in-depth examination of the preprocessed data was conducted via principal component analysis (PCA), facilitated by applications of the “FactoMine” and “factoextra” R packages.

### Analysis of differentially expressed genes and construction of the WGCNA network

The “limma” package, also a function of R, was utilized for screening differentially expressed genes (DEGs) within the IS patient pool and control group in the combat dataset. Threshold values for DEG identification were stringently established at *P* < 0.05 and |log2 fold change (FC)| ≥ 1, respectively. Following this procedure, the identified DEGs of blood samples were introduced for WGCNA using the “WGCNA” package in R (22).

Upon conducting the logarithmic transformation of expression profiles, the hclust function guided the clustering of DEGs into comparable modules. The enable WGCNA Threads function directed multithreaded operations juxtaposed with a scatter plot to distinguish the optimal threshold. In contrast, the urodynamic function was applied to meticulously analyze the gene hierarchical clustering tree, resulting in co-expression modules. Afterward, co-expression modules exhibiting close resemblance (r > 0.75) were grouped.

### Function enrichment of Gene Ontology and Kyoto Encyclopedia of Genes and Genomes and gene set enrichment analysis

The lightgreen module, which included 51 genes of WGCNA results, was considered the platelet-related module. We used the enrichGO and enrichKEGG functions of the “ClusterProfiler” package in Bioconductor (http://bioconductor.org/packages/release/bioc/html/clusterProfer.html) to perform Gene Ontology (GO) and Kyoto Encyclopedia of Genes and Genomes (KEGG) enrichment analysis on genes in the lightgreen module. Specifically, the identification of biological processes (BPs), cellular components (CCs), molecular functions (MFs), and KEGG pathways under the human genome was performed to identify platelet-related BPs and pathways. The *P* value cutoff was 0.05. To determine whether there were any remaining platelet-relevant gene (PRG)-enriched pathways in other modules, the expression profiles of all DEGs were subjected to gene set enrichment analysis (GSEA), and platelet-related GO entries or KEGG pathways and their enriched genes were screened out with adjusted *P* < 0.05 through GSEA function in the “ClusterProfiler” package.

### Construction and analysis of protein–protein interaction

STRING database (https://cn.string-db.org/) was used to conduct the protein–protein interaction (PPI) of 66 genes in platelet-related GO entries or KEGG pathways and the module. Organisms were chosen “Homo sapiens,” the minimum required interaction score corresponding to “high confidence (0.700)” and the “tsv” format of the result and then output into Cytoscape 3.9.0. Then, to identify highly interconnected gene modules, the “MCODE” plugin of Cytoscape was used according to the “K-core>2.” Hub genes were detected with the “cytiHubbvba” plugin of Cytoscape according to their network features. Hub genes and genes in core modules were merged, and duplicates were removed. Lastly, the subsequent establishment of the PPI biological function co-expression network of PRGs was realized by GeneMANIA (http://www.genemania.org/).

### Screening of diagnostic biomarkers via machine learning

Least absolute shrinkage and selection operator (LASSO) was used in this study to screen for significant platelet-associated prognostic genes (PAPGs). After removing genes with 0 coefficient, the “glmnet” package in R was used to perform LASSO and identify genes significantly associated with IS and control samples. The formula for calculating the LASSO risk score is as follows: Risk score = (ExpressionGENE1 × CoefficientGENE1) + (ExpressionGENE2 × CoefficientGENE2) +…+ (ExpressionGENEn × CoefficientGENEn) (23). LASSO coefficient maps and curves are presented in R using the plot function.

### Validation of diagnostic efficacy of signature genes in IS

The samples in the combined expression matrix of GSE16561 and GSE22255 were divided into high-risk groups and low-risk groups according to the risk score of LASSO. The risk scores of high- and low-risk groups for each gene set and the expression levels of PAPGs in IS patients and controls were presented as box plots using the “ggplot2” package in R. We further evaluated its diagnostic potential in GSE16561 and an outside validation group, GSE22255, respectively. The “ROCR” package in R was used to perform the receiver operating characteristic (ROC) curve and evaluate the diagnostic potential of the PAPGs. The value of the area under the curve (AUC) greater than 0.7 indicated favorable diagnostic performance.

### Immune cell infiltration

The Cell-type Identification By Estimating Relative Subsets Of RNA Transcripts (CIBERSORT), a computational method that can analyze the expression profile matrix by vector regression, can identify 22 human immune cell subtypes. We used the “CIBERSORT” package in R to explore the differences in immune cell composition between IS and normal patients. The “corrplot” package was used to draw the correlation between immune cell composition and PAPGs by the Spearman method.

### Construction of mRNA–miRNA interaction network

Two analytical tools, MiRTarBase and miRWalk database, were used to predict the pivotal miRNAs targeted by PAPGs. The results of the two databases were taken from the intersection of the miRNAs to screen out the miRNAs targeting more than two genes as the retention.

### Drug discovery in Connectivity Map

Connectivity Map (CMap) is an open database (https://www.broadinstitute.org). We used to predict small molecule compounds that may induce or reverse the altered expression of PAPGs in cell lines and to identify connections between potential drugs that share chemicals, physiological processes, and mechanisms of action (24). It came into service to screen potential drugs according to PRGs in IS patients.

### Molecular docking analysis

The obtained potential antiplatelet drugs of IS patients were docked with six proteins in PAPGs. Molecular docking was performed using AutoDockTools 1.5.6 and AutoDock Vina 4.2. Briefly, the docking is as follows. Firstly, the core compound structure files (mol2 format) were downloaded from the PubChem database. ChemDraw was used to minimize the structure energy and convert the structure into a 3D structure. Then, the target crystal structure was obtained from the PDB database (https://www.pdb.org/x) and imported into PyMOL 1.7.2.1 (https://pymol.org/2/x) for dehydration and hydrogenation for ligand separation. Docking grid boxes were subsequently constructed in AutoDockTools 1.5.6 at the active site of each target protein and then saved in pdbqt format. Molecular docking of putative targets and active compounds using AutoDock Vina 4.2 and evaluating free binding energies. Finally, visualize and analyze the interaction and critical patterns between drugs and proteins using PyMOL and Discovery Studio 2020.

### Materials

Linseed oil (purity ≥99%) was purchased from Aladdin (Shanghai, China). Ciprofibrate (molecular weight = 289.15; purity >98%) was obtained from Aladdin (Shanghai, China). Enteric-coated aspirin was purchased from Bayer Healthcare Co. Ltd. (Beijing, China). Rat ELISA kits for 6-keto-prostaglandin F1α (6-keto-PGF1α), thromboxane B2 (TXB2), tissue plasminogen activator (tPA), and plasminogen activator inhibitor (PAI) were acquired from RuiDaHengHui Science & Technology Development Co., Ltd. (Beijing, China). Human assay kits for fibrinogen (FIB) content were obtained from Mantino Medical Devices Co., Ltd. (Changchun, China). TRIzol (155960-18) was purchased from Ambin (Beijing, China). Triphenyltetrazolium chloride (TTC) Solution (2%) was purchased from Solarbio (Beijing, China). Red Blood Cell Lysis Buffer (C3702) and DEPC water (R0022) were provided by Beyotime (Shanghai, China). PrimeScript™ RT Master Mix (Perfect Real Time) (RR036A) and TB Green Premix Ex Taq II (Tli RNaseH Plus) (2X) (RR036A) were purchased from Takara (Beijing, China).

### Animals

In this experiment, healthy male Sprague-Dawley (SD) rats (230–270 g) were purchased from Beijing Vital River Laboratory Animal Technology Co., Ltd. (Beijing, China). All of the SD rats were adapted to ventilated cages (temperature: 20°C–25°C, relative humidity: 30%–50%) under a 12-h light/dark cycle and were given free access to food and water. All animal care and experimental protocols were approved by the Institutional Animal Care and Use Committee of the Chinese Academy of Medical Sciences & Peking Union Medical College (SYXK 2023–0008). All efforts were made to minimize the number of animals used and to ensure minimal suffering.

### Medial cerebral artery occlusion/reperfusion model

The SD rats were anesthetized with isoflurane (4% for initiating anesthesia in a chamber and 1.5% for maintaining anesthesia afterward), and the cerebral I/R was induced by medial cerebral artery occlusion/reperfusion (MCAO/R) operation. In a nutshell, the suture occlusion technique was used to occlude the middle cerebral artery, and the lines were then removed from the common carotid artery after 120 min (25). The sham-operated rats suffered from the same procedure apart from the sutures inserted into the internal carotid artery. A heating pad (Sunbeam, USA) was used to keep the body temperature of rats at 37°C ± 0.5°C. The groups of animals were blinded; the researchers did not know to which group each animal was assigned.

### Drug treatment

The drugs were dissolved in 5% carboxymethylcellulose sodium before administration. All of the drugs were chronically delivered into bodies by intragastric (i.g.) administration. The rats were randomly assigned to five experimental groups: the sham group, the MCAO/R group, the ciprofibrate + MCAO/R group, the linseed oil + MCAO/R group, and the aspirin + MCAO/R group. Linseed oil (3 mL kg^-1^), ciprofibrate (7.5 mg kg^-1^), or aspirin (positive control drug, 30 mg kg^-1^) were given 14 days before MCAO surgery.

### TTC staining

TTC staining was conducted 24 h after I/R to determine whether the cerebral infarction model was successfully established and the reduction of cerebellar infarction volume in the treatment groups. The rats’ brains were frozen at -20°C for 20 min, cut into 2-mm coronal slices, placed in TTC staining solution (2%), incubated at 37°C for 15 min, and overnight in 4% paraformaldehyde.

### Enzyme-linked immunosorbent assay

Plasma was collected from each sample. The expression level of FIB, 6-keto-PGF1α, TXB2, t-PA, and PAI was assessed using enzyme-linked immunosorbent assay (ELISA) kits according to the previous method (26).

### Blood total RNA extraction and real-time quantitative polymerase chain reaction

Real-time quantitative polymerase chain reaction (RT-qPCR) was used to assess mRNA expression. First, total RNA was isolated from arterial blood nucleated cells using TRIzol and red blood cell lysate. Complementary DNA was synthesized using PrimeScript™ RT Master Mix. PCR primer sequences are shown in **Supplementary Table S1**. The prepared cDNA, GAPDH, and TB Green were used as a template and reference for RT-qPCR reactions on LightCycler96 Real time PCR System (Roche, USA). Amplification conditions were as follows: 95°C for 5 min, 95°C for 10 s, 60°C for 30 s, and 72°C for 30 s, for a total of 35 cycles. Eventually, the relative mRNA expression was analyzed using the 2^−ΔΔCq^ method [ΔCq = Cq (target gene)-Cq (reference gene)] (27).

### Statistical analysis

The bioinformatics analysis was conducted with R software (Version 4.3.0). GraphPad Prism9.5 was used for statistical analysis in biological experiments. Data were analyzed using Student’s t-test or one-way ANOVA, followed by Tukey’s test or two-way ANOVA and Bonferroni’s multiple comparison test to determine whether the data were normally distributed. *P* < 0.05 was deemed statistically significant in all cases. ImageJ 1.44p software (National Institutes of Health, Bethesda, MD, USA) was used to quantify the cerebral infarct area.

## Results

### Experimental design

**Figure 1:**
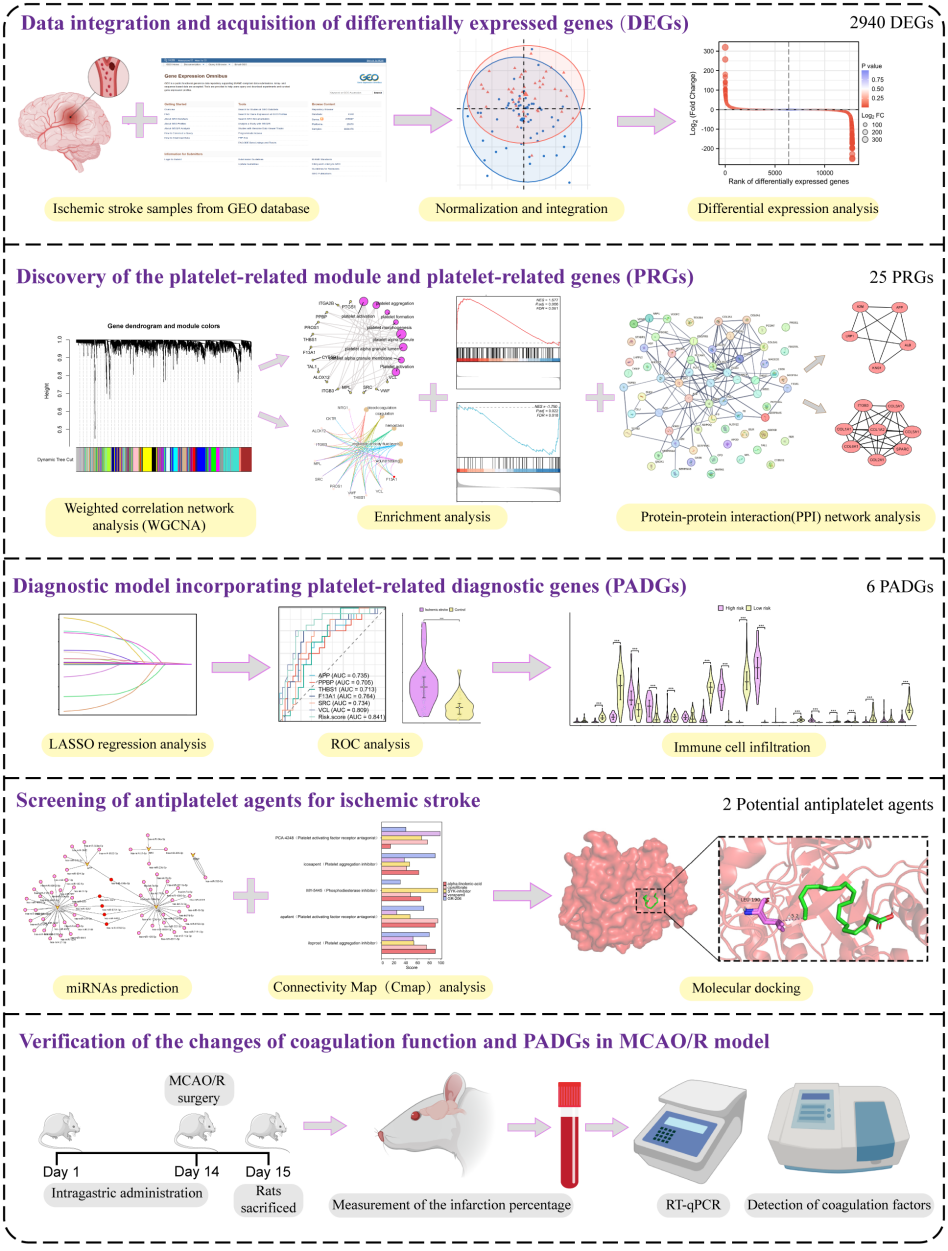
The experimental technical road map of the whole essay.

The study’s workflow is depicted in **Figure 1**, which outlines each step of the methodology. Initially, the data were processed to DEGs. Two datasets, GSE22255 and GSE16561, were amalgamated and normalized to create a comprehensive gene expression profile. The DEGs were subsequently applied to the WGCNA and PPI network to identify platelet-related modules and corresponding genes. A total of 25 PRGs were distinguished. Following this, the LASSO algorithm formulated a platelet-related diagnostic model, including six diagnostic molecular markers. Potential IS-related antiplatelet drugs were forecasted, and their molecular binding to platelet-related diagnostic genes (PADGs) was illustrated. The possibility of miRNAs that could modulate the expression of these markers was also projected. The final step involved validating the changes in the expression of these proposed diagnostic markers in rat blood samples pre- and post-IS. Additionally, predictions related to the potential drug’s effectiveness in preventing cerebral infarction and enhancing coagulation capacity were verified.

### Data processing and DEG identification

**Figure 2:**
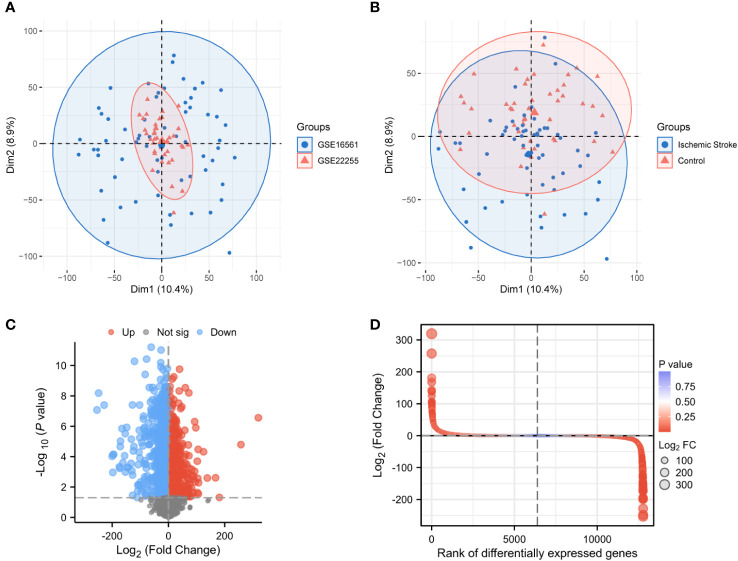
Normalization of the dataset and acquisition of differentially expressed genes. PCA plots for GSE16561 and GSE22255 samples **(A)**. PCA plots for IS and control samples **(B)**. Volcano plot of DEGs in IS patients and healthy individuals (|logFC| > 2, *P* < 0.05) **(C)**. Differential fold plot of DEGs between IS patients and healthy individuals **(D)**. PCA, Principal components analysis; DEGs, Differentially expressed genes; IS, Ischemic stroke; logFC, Log fold change.

PCA between the two datasets and between IS patients and controls showed that normalized GSE16561 covered GSE22255, with apparent differences in samples between IS patients and management (**Figures 2A, B**). After differential analysis of the expression profile of GSE22255 and GSE16561 combination, 2,940 DEGs between IS patients and controls were obtained, of which 1,753 upregulated genes were shown in red, 1,187 downregulated genes were shown in blue, and the screening condition was *P* < 0.05 and |logFC| > 1(FC, fold change; adj.*P*: adjusted *P* value), black dots in the volcano plot represent undifferentiated genes (**Figures 2C, D**).

### Establishment of the WGCNA network and identification of the platelet-related module

**Figure 3:**
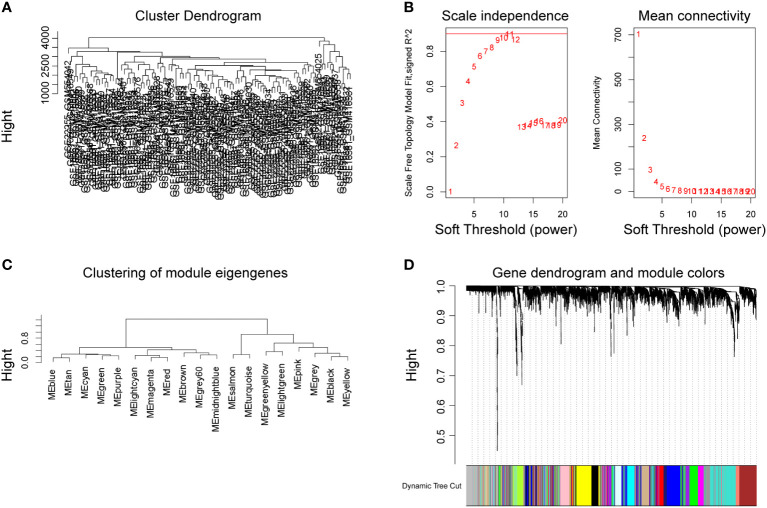
Establishment of WGCNA network of DEGs. Cluster tree of 102 samples after clipping **(A)**. Analysis of the scale-free exponent and the average connectivity of each soft threshold, the red line indicates the minimum soft threshold of 9 for constructing the scale-free network **(B)**. Clustering of modular characteristic genes for all DEGs **(C)**. Gene dendrogram for all DEGs **(D)**.

After removing missing values in the expression profile, we detected heterogeneity in each sample by hierarchical clustering trees, set the cut height to 4,500, used the “cutreeStatic” function to exclude outliers in the study, and included all samples after the cut in the subsequent research. A total of 12,773 genes and 102 representatives from the gene expression matrix were used for WGCNA analysis (**Figure 3A**). The “pickSoftThreshold” function guided the multithreaded work and filtered the soft thresholds (**Figure 3B**). The optimal soft threshold was set to 9. Based on the weighted network and the mutual co-expression of genes, we performed a hierarchical cluster tree analysis to cluster the genes that can interact with each other to generate modules with the most similar expression. Based on their expression profiles, a total of 18 modules were obtained (**Figure 3C**). Dendrogram branching indicated that the genes in each module were highly heterogeneous (**Figure 3D**).

**Figure 4:**
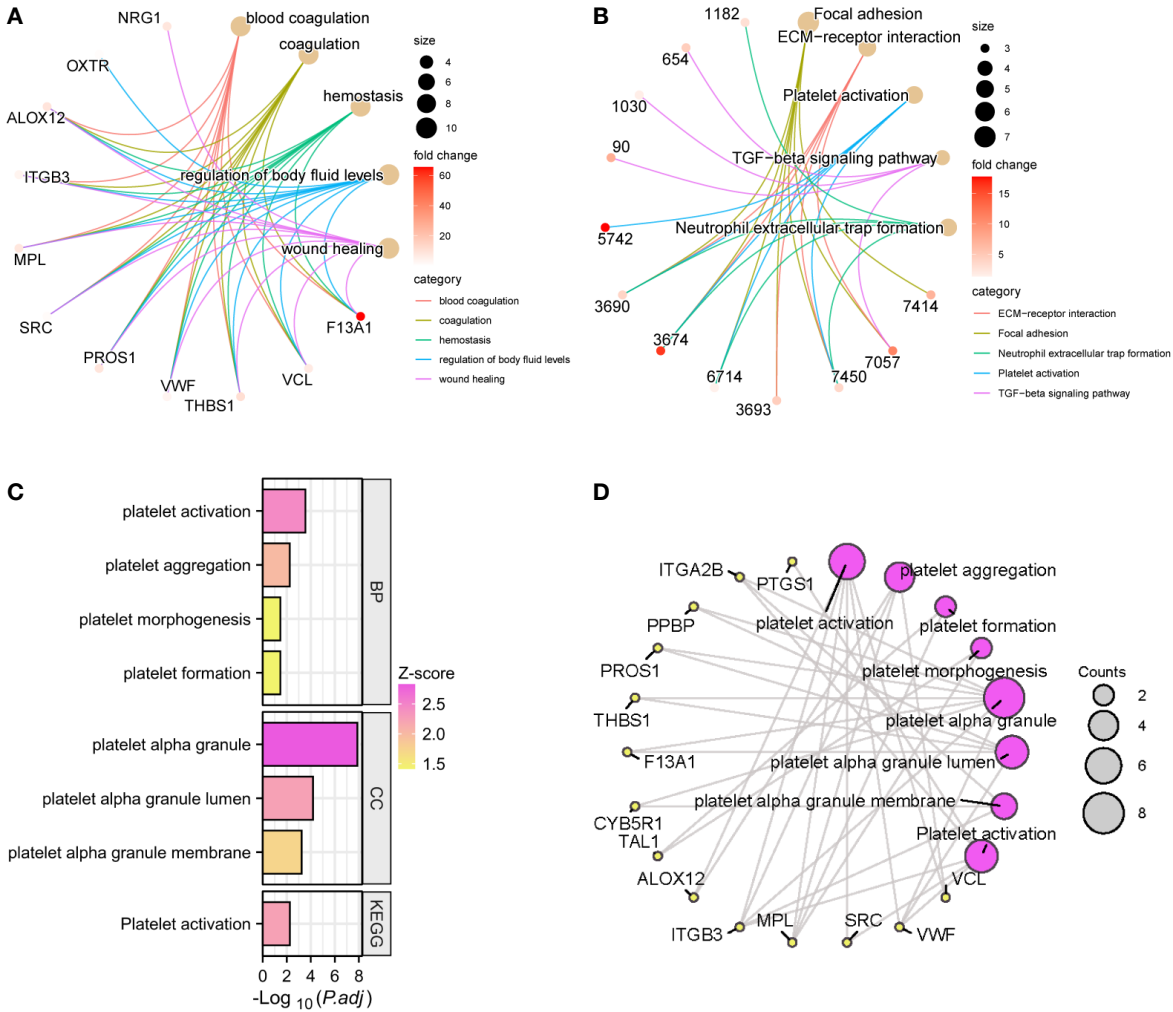
GO and KEGG enrichment analysis of the lightgreen module. The top 5 GO entries **(A)** and KEGG pathways **(B)** of enriched genes and their enriched targets were identified. Platelet-related enrichment analysis entries **(C)** and their corresponding targets **(D)** in the lightgreen module.

We performed GO and KEGG enrichment analysis on the 18 modules obtained by WGCNA, respectively. Module 7, also known as the lightgreen module, was designated as the platelet-related module due to the significant enrichment of pathways and biological processes associated with platelets and coagulation exclusively within this module (**Supplementary Table S2**). It consisted of 51 genes, among which 50 genes demonstrated upregulation, while one gene exhibited downregulation within the expression profile of IS patients. GO and KEGG enrichment analysis was performed on the genes in this module. The top 5 enriched genes in the biological processes of GO were blood coagulation, coagulation, hemostasis, regulation of body fluid level, and wound healing (**Figure 4A**). KEGG’s top 5 enriched pathways included platelet activation, TGF-β signaling pathway, etc. (**Figure 4B**). In addition, there are many biological processes and molecular functions related to platelets in this module, including platelet aggregation, platelet morphogenesis, platelet formation, platelet α-granule, platelet α-granule lumen, and platelet α-granule membrane (**Figure 4C**). The relationships between these platelet-related entries and the 14 genes enriched were plotted as a network (**Figure 4D**).

### Analysis of GSEA and PPI networks and integration of PRGs

**Figure 5:**
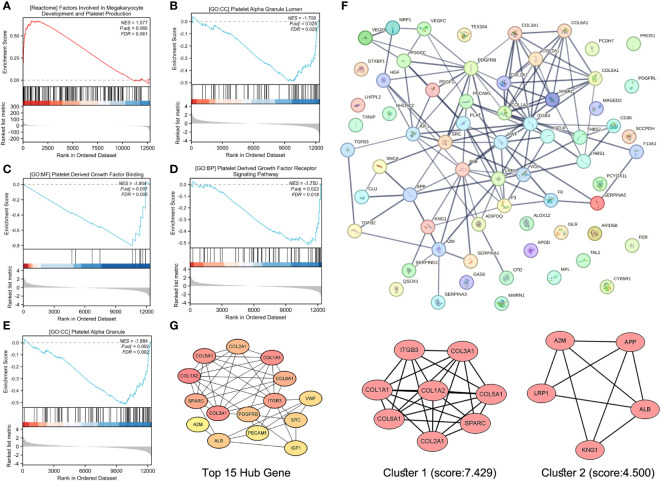
Screening and recruitment of PRGs. GSEA of platelet-related KEGG gene set **(A)**, platelet-related GO.CC gene set **(B, E)**, platelet-related GO.MF gene set **(C)**, and platelet-related GO.BP gene set for all genes **(D)**. Protein–protein interaction (PPI) analysis of platelet-associated gene sets **(F)**. The Top 15 Hub gene of the PPI network, and from yellow to orange to red, genes get higher and higher scores, the top 2 modules of this network, and K-core = 2, module 1 score = 7.429, module 2 score = 4.500 **(G)**.

Although the lightgreen module was identified as the most platelet-related module, GSEA was performed for all expressed genes to avoid missing PRGs in stroke patients in other modules. Indeed, numerous platelet-related gene sets were identified and were enriched for factors involved in megakaryocyte development and platelet production, platelet alpha granules, platelet-derived growth factor binding, platelet-derived growth factor receptor signaling, and platelet alpha granules (**Figures 5A-E**).

Deletion of duplicate values from these platelet-related gene sets resulted in 65 genes that were entered into STRING for protein–protein interaction analysis, with a confidence of greater than 0.700 (**Figure 5F**). The “cytohubba” plugin in Cytoscape 3.9.0 analyzed the above PPI network and screened the top 15 genes (**Figure 5G**). The “MCODE” plug-in was also used, and the genes with a degree value less than 2 were trimmed. Node Score Cutoff was selected as 0.2, K-core as 2, and Max. Depth was chosen as 100 to cluster the remaining genes. A total of two modules were obtained: module 1 had 8 nodes and 26 edges with a score of 7.429 (**Figure 5G**), and module 2 had 5 nodes and 9 edges with a score of 4.500 (**Figure 5G**). Finally, we summarized 15 Hub genes, 8 genes obtained by clustering 14 genes enriched in platelet-related entries in 14 lightgreen, and 25 genes were confirmed as PRGs (**Supplementary Table S3**).

### Identification of PADGs and validation of diagnostic models

**Figure 6:**
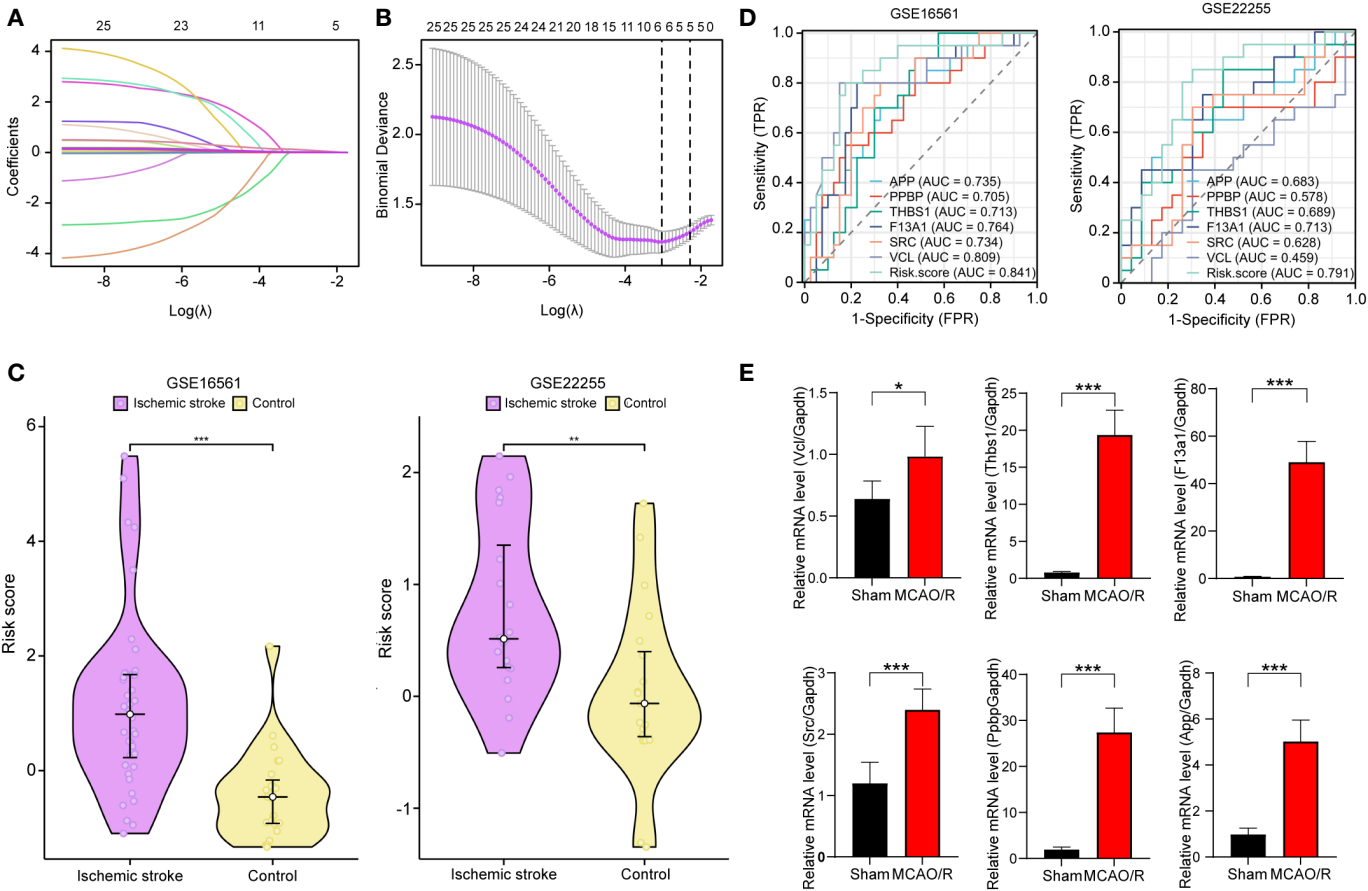
Development and validation of platelet-related diagnostic models. Distribution of LASSO regression coefficients for PADGs **(A)**. The parametric plot of LASSO regression for PRGs **(B)**. Risk scores of platelet-related diagnostic models for IS patients and healthy people in GSE16561 and GSE22255 **(C)**. ROC curve analysis of individual factors and diagnostic models of GES16561 and GSE22255 **(D)**. Relative mRNA levels of Vcl, Thbs1, F13a1, Src, and App in sham and MCAO/R groups **(E)**. (* *P* < 0.05, ** *P* < 0.01, *** *P* < 0.001.).

We obtained the expression data of 25 PRGs from the combined expression profiles of GSE16561 and GSE22255 as the training set. We then applied the LASSO algorithm to derive coefficient profiles (**Figure 6A**) and partial 191 likelihood deviations (**Figure 6B**) using the “glmnet” package in R. From these analyses, we identified six labels with non-zero coefficients, namely, APP, THBS1, F13A1, SRC, PPBP, and VCL; these tags were used to construct the LASSO regression model and were identified as PADGs. The risk score formula was as follows: riskScore = 0.032435376×ExpressionMAPK3 + 0.000297749 × ExpressionPPBP + 0.015633334 ×ExpressionTHBS1 + 0.001329085× ExpressionF13A 1 + 0.118914685 × ExpressionSRC +0.015304202 × ExpressionVCL. The expression matrices of PRGs from each dataset of GSE16561 and GSE22255 were selected as two separate validation sets with forgeneralized cross-validation of the risk score model. Patients with IS in each cohort had significantly higher risk scores than the control samples (**Figure 6C**). ROC analysis was subsequently used to determine the diagnostic potential of our model. In the two validation sets, the AUC of the total risk screen was 0.841 and 0.791, respectively, which was larger than the AUC of any single variable screened by the LASSO model in the validation set (**Figure 6D**). Additionally, we conducted external validation using mRNA samples from the peripheral blood individuals affected by stroke and healthy individuals within the GSE202709 dataset. The risk score within the stroke population group was notably higher than that within the healthy group (**Supplementary Figure S1A**), and the ROC analysis illustrated an AUC value of 0.917 (**Supplementary Figure S1B**). This indicates that the diagnostic potential of the risk score model is greater than that of any single PADG.

In addition, to test the reliability of this model, we performed cerebral I/R in rats. The RT-qPCR findings showed that the relative mRNA levels of VCL, THBS1, F13A1, SRC, and APP in the MCAO/R group’s blood rose significantly (*P* < 0.05 or *P* < 0.0001) (**Figure 6E**). This trend aligns with the expression pattern these biomarkers showed in human peripheral blood in previous screenings.

### Immune cell infiltration of PADGs

**Figure 7:**
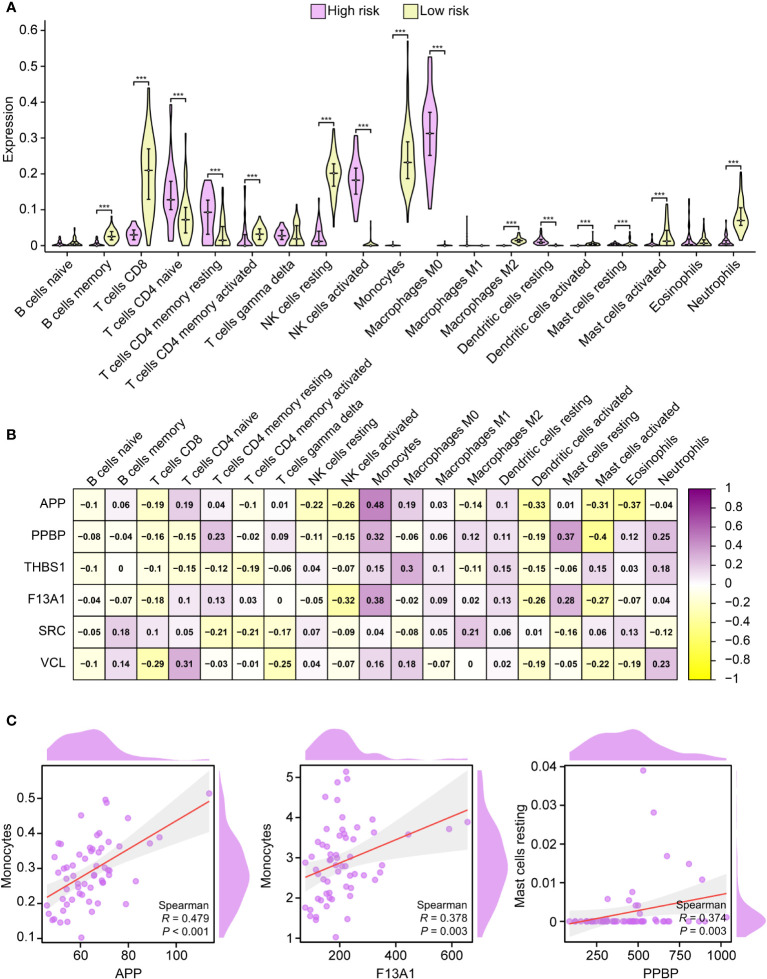
Immune infiltration analysis. Correlation between high-risk and low-risk groups with 19 immune cells **(A)**. Correlation analysis between PADGs and 19 kinds of immune cells **(B)**. Scatter plot of association between APP and monocytes, F13A1 and monocytes, and PPBP and resting mast cells **(C)** (* *P* < 0.05, ** *P* < 0.01, *** *P* < 0.001).

CIBERSORT represents a deconvolution computational algorithm designed to estimate the proportions of 22 immune cells within a given tissue based on the gene expression derived from RNA sequencing (28, 29). In this study, we employed the “CIBERSORT” tool within the R environment to assess the immunological profiles of peripheral blood samples obtained from both high-risk and low-risk individuals. Populations at high risk had higher naive CD4 T cells, resting CD4 memory T cells, activated CD4 memory T cells, activated natural killer (NK) cells, macrophages M0, and resting dendritic cells compared with the population at low risk. On the contrary, memory B cells, CD8 T cells, resting NK cells, monocytes, macrophages M2, activated mast cells, and activated neutrophils were lower (**Figure 7A**). All PADGs were positively correlated with monocytes and resting dendritic cells and negatively correlated with naive B cells and activated NK cells. APP and SRC were negatively correlated with neutrophils, while PPBP, THSB1, F13A1, and VCL were positively correlated with neutrophils. Only VCL was negatively correlated with macrophage M1 (**Figure 7B**). In addition, the correlation coefficients between APP and monocytes, F13A1 and monocytes, platelet basic protein (PPBP), and resting mast cells were large. All *P* values were less than 0.05 (**Figure 7C**). APP and F13A1 may cause changes in the immune microenvironment by regulating monocytes after IS.

### Screening of small-molecule drugs

**Figure 8:**
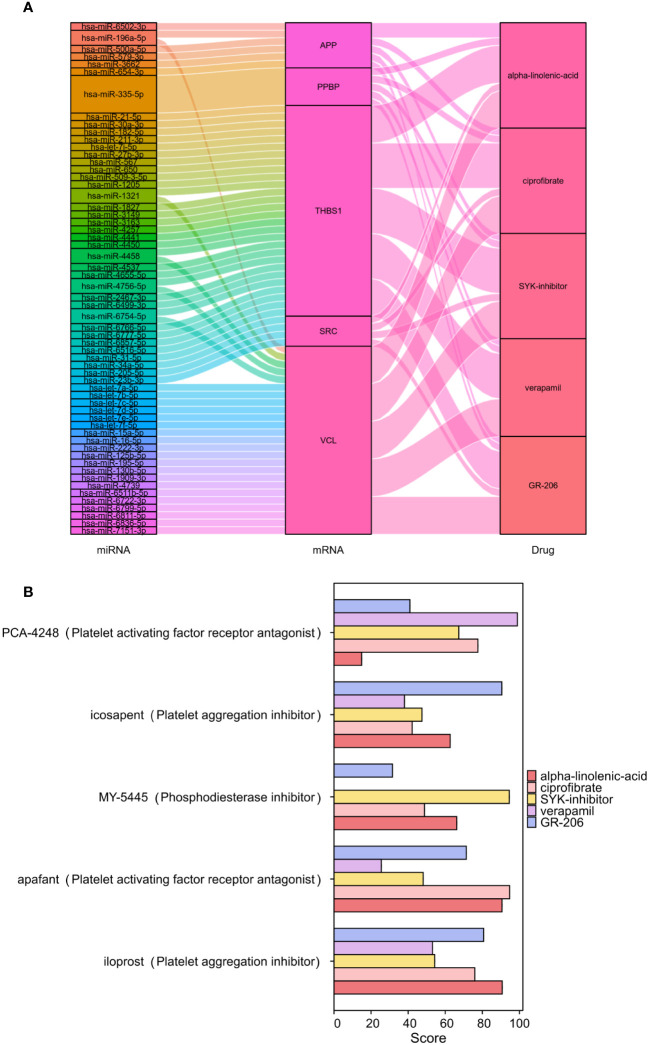
Screening of antiplatelet-related small-molecule drugs in IS. Construction of the miRNA-mRNA-drug network **(A)**. Scoring of the pharmacological similarities between the top 5 compounds and platelet inhibitors **(B)**.

The CMap was used to compare the reference gene expression profiles after drug treatment according to the upregulated or downregulated differential genes to find possible small-molecule drugs. The upregulated genes in the 22 PRGs were used to predict potential drugs, and 30 antiplatelet-related drugs were screened out, as shown in **Supplementary Table S4**. We employed miRWalk 2.0 software to conduct mRNA-miRNA analysis on six genes within PADGs (**Supplementary Figure S2A**). Subsequently, 59 miRNAs were subjected to enrichment analysis using GeneCodies, revealing significant enrichment in biological functions pertaining to protein phosphorylation, negative regulation of transport processes, and signal transduction. Additionally, the enriched biological pathways encompassed signaling events in the FoxO signaling pathway, lipid and atherosclerosis, and the AMPK signaling pathway (**Supplementary Figures S2B, S2C**). The first five drugs (alpha-linolenic acid, ciprofibrate, SYK inhibitor, verapamil, and GR-206) were selected to construct a miRNA-mRNA-drug network (**Figure 8A**). The top 5 drugs were entered into the CMap touchstone database, which facilitates the exploration of connections between the genetic alterations of genes and the drug signatures, in order to identify compounds with similar pharmacological effects. Each predicted drug exhibited more than 90% similarity to at least one existing antiplatelet agent based on alterations in gene expression profiles: alpha-linolenic acid with platelet-activating factor receptor antagonist, GR-206 with platelet aggregation inhibitor, SYK inhibitor with phosphodiesterase inhibitor, verapamil with platelet growth factor receptor antagonist, ciprofibrate with platelet growth factor can be seen. Receptor antagonists are highly similar (**Figure 8B**).

### Molecular docking of compounds with PADGs

**Figure 9:**
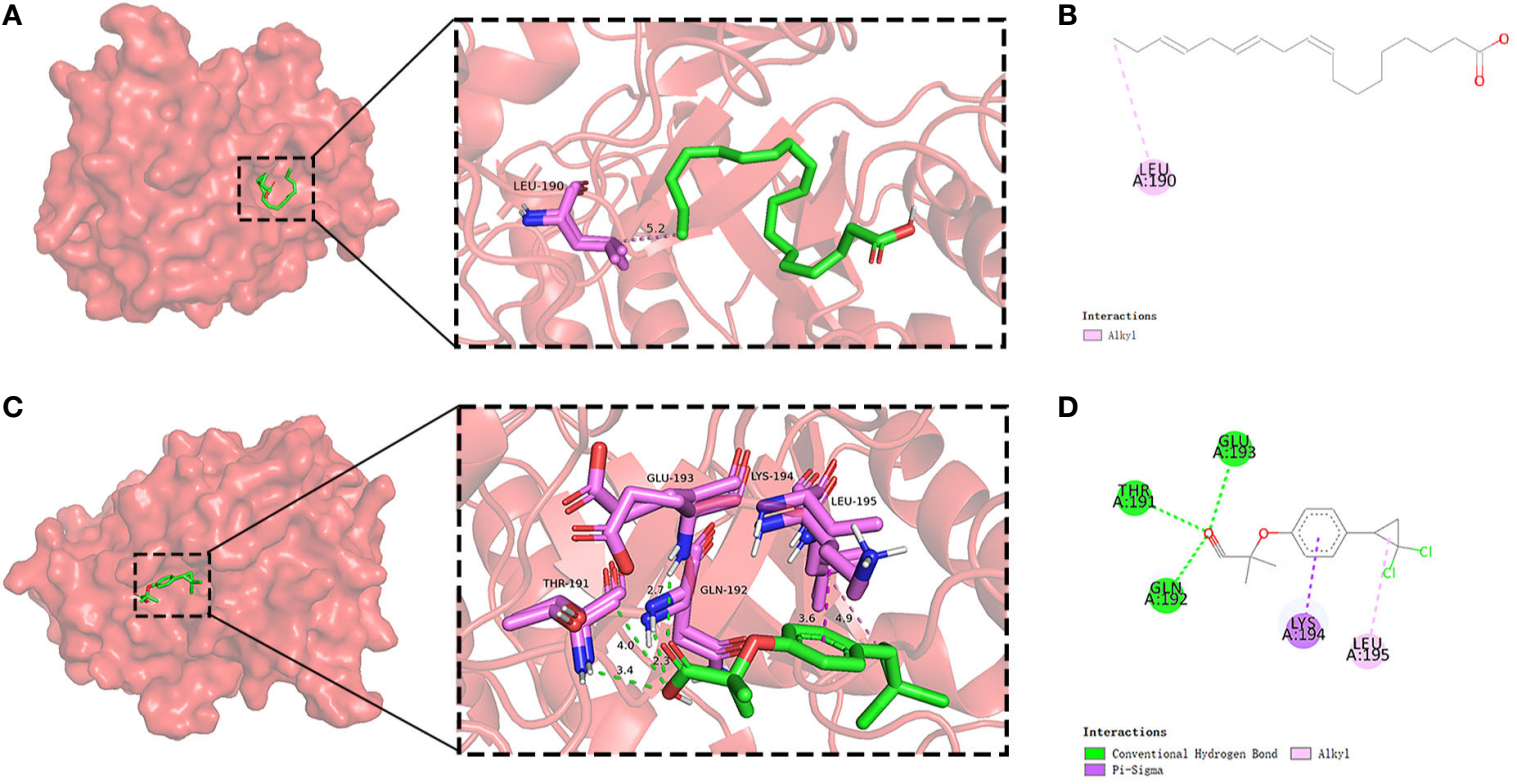
Molecular docking of SRC and small-molecule drugs. Docking situation **(A)** and interaction **(B)** of SRC and alpha-linolenic acid. Docking situation **(C)** and interaction **(D)** of SRC and ciprofibrate.

In light of the structural formula of SKY inhibitors is not single, alpha-linolenic acid, and ciprofibrate were selected for molecular docking with six PADGs in Autoduck, each with 20 conformations. A total of 18 groups were docked, and the lowest binding energy of the docked conformation in each group was selected and shown in **Table 2**. A binding energy of less than -5 indicates good docking in nine groups. The ligands in the other three groups were all alpha-linolenic acid, and the absolute values of binding energies were low. The binding of SRC to these two compounds to other proteins suggests that SRC may be an essential target for the pharmacological effects of these drugs. Subsequently, we conducted visualizations of the docking process involving SRC and these two compounds, employing Discovery Studio to illustrate a 2D map demonstrating the interaction between the receptor and the ligand. From the range of conformations, we selected the conformation displaying the best overlap for further analysis of the binding interaction. Specifically, α-linolenic acid was observed to form an alkyl bond with the 190th amino acid (LEU) of the A-chain of SRC (1us0). Conversely, ciprofibrate engaged in hydrogen bonds with the 191st (THR), 192nd (GLN), and 193rd (GLU) amino acids of the A chain of SRC (1us0), while establishing πσ bonds with the 194th amino acid (LYS) of the A chain. Additionally, it formed an alkyl bond with the 195th amino acid (LEU) of the A chain, as illustrated in **Figures 9A-D**.

**Table 2:** Docking binding energy of PADGs and small-molecule drugs.

Rank	Target	PBD ID	Compound	PubChem ID	Affinity (kcal/mol)
1	SRC	1us0	Ciprofibrate	2763	-9.383
2	SRC	1us0	Alpha-linolenic acid	5280934	-7.815
3	PPBP	7pud	Ciprofibrate	2763	-7.527
4	F13A1	4kty	Alpha-linolenic acid	5280934	-6.185
5	APP	5z6d	Ciprofibrate	2763	-6.000
6	VCL	4ln2	Ciprofibrate	2763	-5.985
7	F13A1	4kty	Ciprofibrate	2763	-5.885
8	THSB1	2erf	Ciprofibrate	2763	-5.339
9	PPBP	7pud	Alpha-linolenic acid	5280934	-5.226
10	APP	5z6d	Alpha-linolenic acid	5280934	-4.655
11	VCL	4ln2	Alpha-linolenic acid	5280934	-4.152
12	THSB1	2erf	Alpha-linolenic acid	5280934	-3.445

### Verification of the changes of coagulation function in rat MCAO/R model

**Figure 10:**
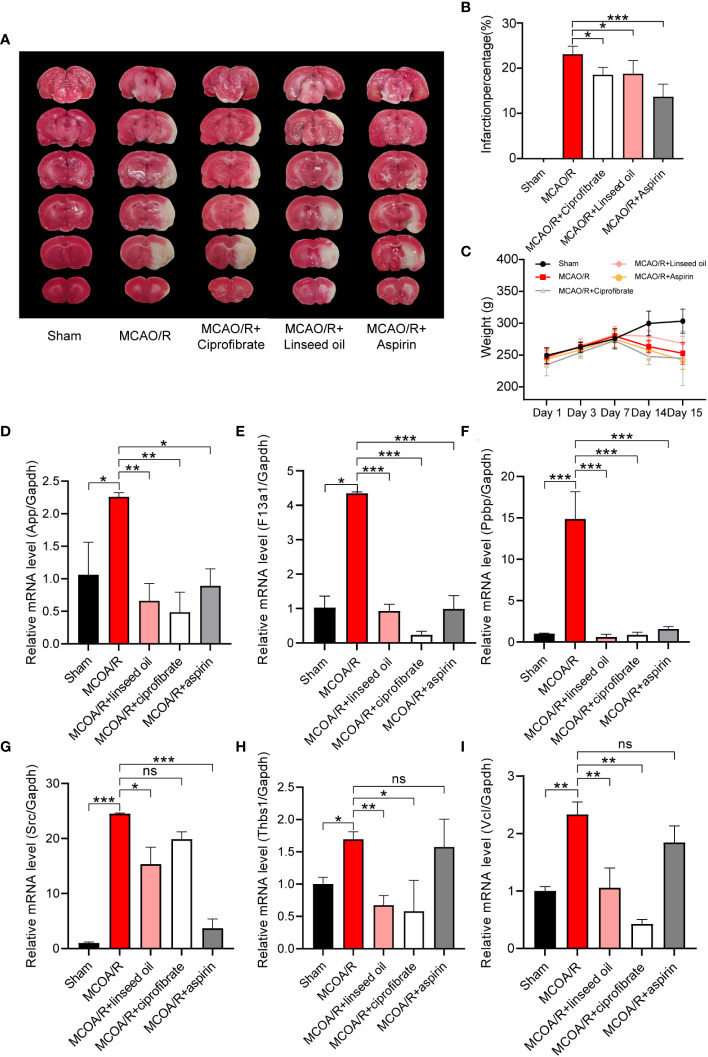
Verification of the changes in coagulation function and diagnostic marker expression. TTC staining of brain slices **(A)** and the infarction percentage **(B)** statistics. The changes in body weight of rats in each group were calculated from the first day of administration to the 15th day before sacrifice **(C)**. Relative mRNA levels of App, F13a1, Ppbp, SRC, Thbs1, and Vcl in sham, MCAO/R, MCAO/R + linseed oil, MCAO/R + ciprofibrate, and MCAO/R + aspirin groups **(D-I)**. (* *P* < 0.05, ** *P* < 0.01, *** *P* < 0.001.).

Based on their highest scores, pharmacodynamic experiments and coagulation factor detection were performed using the top-ranked predicted drugs, ciprofibrate and linseed oil. The linseed oil utilized had an alpha-linolenic acid content of 53%. Results depicted in **Figures 10A, B** show that after I/R, the percentage of infarction stood at 23.10% ± 1.57%. Notably, this was reduced to 18.54% ± 1.44% following the prophylactic administration of ciprofibrate and to 18.76% ± 2.61% after the administration of linseed oil. This reduction was statistically significant when compared with the sham-operation group (*P* < 0.05). However, the body weight observed among the groups did not differ significantly, with the exception of the sham group, as illustrated in **Figure 10C**.

In **Figures 10D–I**, it was observed that the mRNA levels of App, F13a1, Ppbp, SRC, Thbs1, and Vcl exhibited a significant increase subsequent to MCAO induction (*P* < 0.05, *P* < 0.01, or *P* < 0.001). Notably, the two drugs foreseen by CMap—namely, linseed oil (a major component of α-linolenic acid) and ciprofibrate—nearly reversed the MCAO-induced elevation in mRNA levels of these six genes (*P* < 0.05, *P* < 0.01, or *P* < 0.001). Although aspirin did not lead to a significant reduction in the mRNA levels of Vcl and Thbs1 compared with the MCAO/R group, it effectively reversed the mRNA levels of the remaining four genes. These findings align closely with the earlier predicted alterations in gene expression profiles of the drugs.

**Figure 11:**
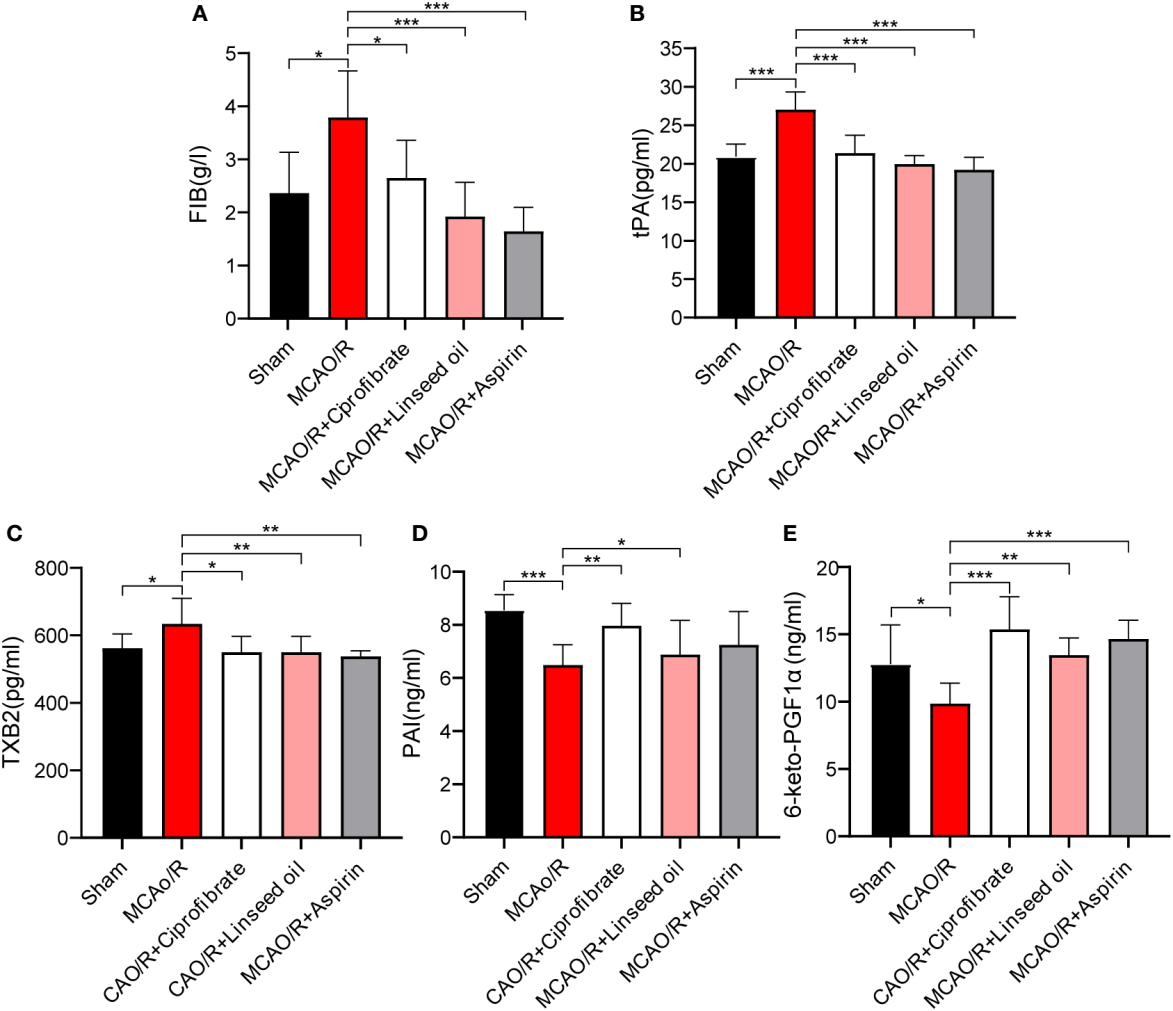
The content of coagulation factors between the different groups. The FIB content of each group **(A)**. The tPA content of each group **(B)**. The TXB2 content of each group **(C)**. The PAI content of each group **(D)**. The 6-keto-PGF1αcontent of each group **(E)**. (* *P* < 0.05, ** *P* < 0.01, *** *P* < 0.001.).

Subsequent figures, specifically **Figures 11A–C**, indicate a significant increase in FIB, tissue tPA, and TXB2 levels in rats after MCAO (*P* < 0.05 or *P* < 0.001). While not as impactful as aspirin in returning the indices to normal levels, both ciprofibrate and linseed oil treatments resulted in substantial reductions in the plasma levels of these factors (*P* < 0.05, *P* < 0.01, or *P* < 0.001, respectively). These substantial trends evidence the valuable preventative impact of these two drugs on the enhancement of coagulation function post-IS by our initial predictions. Contrastingly, in **Figures 11D, E**, the levels of PAI and 6-keto-PGF1α declined significantly after MCAO (*P*<0.05 or *P*<0.001). However, more significant increases were recorded in the ciprofibrate group than in the aspirin group when compared to MCAO/R group (*P*<0.01 or *P*<0.001).

## Discussion

IS represents a gene-associated multivariate and heterogeneous circulatory system aberration characterized by a high mortality rate and protracted functional incapacitation. Prior peer-reviewed empirical investigations have explored the transcriptional profiles presented in the peripheral blood of IS patients or within murine (MCAO) and rat brain tissue to delineate biomarkers and therapeutic targets for IS. Notwithstanding, the DEGs revealed inconsistencies across separate studies (30–32).

The presented study is pioneering in employing a confluence of LASSO, WGCNA, and CIBERSORT algorithms, utilizing platelets as definitional tags to unearth novel biomarkers and diagnostic models germane to IS, grasping the diversity and intricacy of the immune microenvironment, and exploring prospective antiplatelet drugs. The current investigation proposes platelet-linked diagnostic markers in IS, specifically APP, SRC, PPBP, F13A1, VCL, and THSB1. Upon conducting a comprehensive bioinformatics analysis, Wicik Z and colleagues ascertained APP to be firmly tied to collective platelet activity, designating it as one of the genes most susceptible to noncoding regulation in diseases related to platelet reactivity (33), a conclusion in line with our findings. Furthermore, the probability prediction models integrating these PADGs proved highly accurate (with AUC > 0.7) in diagnosing IS within both cohorts.

The enrichment analysis of the 51 DGEs housed in the lightgreen module ascertains these genes’ participation in biological procedures including, but not limited to, “blood coagulation,” “coagulation,” and “hemostasis” following IS. They have salient correlations with the formation, activation, and aggregation of platelets in the bloodstream and platelet α particle genesis and release in the uterine cavity, thereby validating their engagement with IS onset and progression. Embolic stroke is fundamentally anchored in arterial thrombosis, with platelets first observed in blood over 130 years prior and acknowledged as the primary cell type regulating such thrombotic events (34). Post-trauma, platelets discharge contents like thrombin A2 and α particles to mobilize and activate a broader number of platelets, whereupon platelet accumulation occurs at the injury site, leading to primary thrombi formation. Moreover, following reperfusion injury, platelets mediate environmental alterations to the circulatory system, play a role in the detrimental T-cell reactions, and exacerbate and propagate neuroinflammation in I/R injury (35). Therefore, compiling platelet-specific biomarkers as diagnostic indicators or potential therapeutic targets for stroke demonstrates wide-ranging promise.

Our findings mirror the consistent motif in similar scholarly investigations identifying identical pivotal genes. In a significant revelation, SRC was observed to be substantially downregulated poststroke in human brain microvascular endothelial cells (HBMECs), contributing to vascular endothelial cell protection from ischemic and oxygen glucose deprivation/reoxygenation (OGD/R) injury. This was achieved by impairing the SRC signaling trajectory, subsequently realizing gentle inhibitory effects on platelet aggregation (36). Symmetrically, SRC is seen to execute a neuroprotective role in stroke (37). In a sophisticated proteomic analysis by George PM et al., PPBP emerged as a key biomarker in transient ischemic attack (TIA) patients’ serum. Substantial elevations in PPBP serum concentrations were noticed in TIA and minor stroke patients compared to counterparts with migraines and a healthy control group (38). Intriguingly, alterations in these crucial genetic regulators could exacerbate IS severity. Regarding this, it was discovered that the homozygous genotype of the F13A1 204Phe allele precipitously elevated IS risk in young females (39).

In analyzing the consolidated microarray gene expression data, differential landscapes of immune cell types were compared between two cohorts defined as high risk and low risk, utilizing the algorithmic approach provided by CIBERSORT. The analysis engaged 22 immune cell types, leading to the discernment of statistically significant disparities in the constitution of 15 immune cells when contrasting the high-risk group against the low-risk group. An elevation in the prevalence of immune cell groups—naive CD4 T cells CD4, resting CD4 memory T cells, activated CD4 memory T cells, activated NK cells, macrophages M0, and the arrangement of dendritic cells—was significantly noted in the high-risk group relative to that in the low-risk group. Conversely, concentrations of memory B cells, CD8 T cells, resting NK cells, monocytes, macrophages M2, activated mast cells, and neutrophils activated were significantly attenuated.

Significant differences were observed in the proportion of T-cell subsets infiltrating the high-risk group compared to the low-risk group. Notably, the immunomodulatory and hemostatic functions of platelets in IS potentially involve CD4 T cells. Our findings align with established evidence, indicating the pivotal role of CD4+ regulatory T cells in mitigating inflammation and reinstating immune homeostasis poststroke; activated platelets may further influence T-cell function through the secretion of diverse elements like PF4 or serotonin (34, 40). Investigations have revealed that memory CD4 T cells can reduce hemorrhagic transformation in murine IS by binding to platelet P-selectin glycoprotein ligand-1 (41). Moreover, the absence of CD84 on platelets impairs CD4+ T-cell motility and cellular infiltration, consequently reducing thrombus formation and neurological impairment (42). Furthermore, platelet GPIb inhibition diminishes the infiltration of immune cells such as T cells, thus mitigating the local inflammatory response in the ischemic brain (43). In contrast to CD4 T cells, CD8 T-cell infiltration was observed to be lower in the high-risk group following platelet activation compared to the low-risk group. Recent evidence has highlighted that platelet-derived TLT-1 acts as a direct immunosuppressant of CD8+ T cells (44). Platelets can also influence antigen presentation by CD8 T cells; they bind to antigen-specific CD8 T cells through major histocompatibility complex class I (MHC-I) processing and cross-presentation of antigens, thereby regulating CD8+ T-cell numbers, functional responses, and outcomes (45).

Additionally, other immune cells have been demonstrated to be closely associated with the onset or prognosis of IS. Postischemic pharmacological intervention has been shown to augment neuroprotection against ischemic cerebral damage, predominantly by curbing the seepage and activation of NK cells, thereby diminishing infarct dimensions (34). Furthermore, the circulating transfer cell platelets were observed to trigger the downregulation of NK G2D ligands with platelet transforming growth factor-β. This results in the mitigation of NK cell cytotoxicity and their capacity to release IFN-γ (46). Aligning with these findings, our data demonstrate the inverse correlation between activated NK cells and the expression of all six PADGs, in direct contrast with the positive correlation identified with monocytes (28). Monocytes, under specific conditions, tend to form aggregates with platelets in circulation. This phenomenon is frequently observed in patients suffering from cardiovascular and cerebrovascular ailments as a sequela of inflammatory provocation and infection (34). A deeper foray into immune infiltration corroborated the fidelity of our risk stratification model, hence fortifying our confidence therein.

The application of GSEA and PPI network examination using MCODE and CytoHubba plugin tools culminated in identifying an aggregate of 25 platelet-associated genes. Further scrutiny of the correspondent gene expression profile revealed potential antiplatelet therapeutics for IS, alpha-linolenic acid and ciprofibrate. These compounds have the potential to modulate the expression of 22 augmented genes. Alpha-linolenic acid, frequently incorporated into dietary regimens, exerts its effects by mitigating platelet activation, which in turn reduces the presence of pro-inflammatory cells and sickle cell quantities, as evidenced in patients with sickle cell disease (34). It has also been shown to curtail platelet clearance in the reticuloendothelial system in those with atherosclerosis and arterial thrombosis (47). Ciprofibrate, a historically prevalent lipid-lowering medicine, displays significant efficacy in decreasing blood lipid concentrations in patients with hypercholesterolemia (48). When applied in conjunction with aspirin for the management and treatment of patients co-diagnosed with atherosclerosis and hyperlipoproteinemia, an enhanced ability for aspirin to inhibit thromboxane A2 formation and exercise antiplatelet effects has been noted (49). Although these pharmaceutical interventions are currently excluded from declarative clinical guidelines for treating or preempting IS, they have promising platelet inhibitory effects in other thrombotic conditions. Their potential inclusion in the antithrombotic treatment of IS may offer significant financial savings in drug development. Subsequently, we utilized molecular docking to simulate the interaction of these three compounds with PADGs. The majority displayed docking binding energies less than 5, with some—such as SRC—indicating superior docking via traditional hydrogen bonds, hydrophobic bonds, or π bonds, suggesting a possible therapeutic target pertinent to the pharmacological efficacy of these compounds.

Lastly, pharmacodynamic experiments confirmed that both ciprofibrate and alpha-linolenic acid-rich linseed oil could significantly improve the elevation of blood coagulation function after IS and had a significant preventive effect on cerebral infarction. Furthermore, nearly all of them were able to substantially reverse the MCAO/R-induced elevation in mRNA levels of App, F13a1, Ppbp, SRC, Thbs1, and Vcl. Notably, the ability of ciprofibrate to modulate alterations in PAI and 6-keto-PGFα1 after IS was even greater than aspirin’s. This suggests that these two drugs can potentially improve cerebral vascular blockage. RT-qPCR also confirmed a significant increase in the expression of PADGs in blood after IS.

In summary, we identified platelet-related diagnostic markers and established a high-accuracy risk assessment model based on various bioinformatics algorithms and computer-aided drug design methods. Meanwhile, we screened and verified diagnostic markers’ expression and associated drugs’ preventive effects. This study contributes a transformative perspective for the diagnosis approach, prevention, and therapeutic intervention of IS and provides new ideas for the search for antithrombotic drugs with fewer side effects. However, we should underscore the importance of balanced control in managing thromboembolic and hemorrhagic risk when preventing and treating IS. Overenthusiastic thrombolysis, albeit effective in resolving the ischemic event, inadvertently enhances the propensity toward cerebral hemorrhage poststroke. Therefore, identifying the equilibrium point of thrombolysis and hemostasis presents a promising direction for future investigations.

## Conclusion

In summarizing this study, we affirm that our risk assessment model, based on PADGs, specifically APP, THBS1, F13A1, SRC, PPBP, and VCL, presents robust diagnostic capabilities for stroke patients. The noteworthy antithrombus agents—alpha-linolenic acid and ciprofibrate—emerge as potential candidate drugs for preventing and treating cerebral thrombosis post-IS. This underlines a promising milieu for exploring antiplatelet therapy and IS management.

## Data availability statement

Publicly available datasets were analyzed in this study. This data can be found here: https://www.ncbi.nlm.nih.gov/; GSE16561, GSE22255.

## Ethics statement

Ethical approval was not required for the studies on humans in accordance with the local legislation and institutional requirements because only commercially available established cell lines were used. The animal study was approved by the Institutional Animal Care and Use Committee of the Chinese Academy of Medical Sciences & Peking Union Medical College (SYXK 2023–0008). The study was conducted in accordance with the local legislation and institutional requirements.

## Author contributions

YG: Conceptualization, Data curation, Methodology, Writing – original draft, Writing – review & editing. YCL: Methodology, Writing – review & editing. MW: Formal analysis, Writing – review & editing. XD: Writing – review & editing. XS: Project administration, Writing – review & editing. YL: Conceptualization, Funding acquisition, Writing – review & editing. XBS: Formal analysis, Funding acquisition, Resources, Writing – review & editing.

## Supplementary material

Supplementary Figure 1 Validation of platelet-related diagnostic models in GES202709. Risk scores of platelet-related diagnostic models for IS patients and healthy people in 202709 **(A)**. ROC curve analysis of individual factors and diagnostic models of GES202709 **(B)**.

Supplementary Figure 2 Prediction and enrichment analysis of PADGS-associated miRNAs. Construction of the PADGS-related miRNA-mRNA network **(A)**. The top 10 items in GOBP, CC, and MF **(B)** and the top 30 enriched pathways **(C)** in KEGG enrichment analysis of miRNAs.

## References

[1] Kuriakose D Xiao Z. Pathophysiology and treatment of stroke: present status and future perspectives. Int J Mol Sci (2020) 21(20):7609. doi: 10.3390/ijms21207609 33076218 PMC7589849

[2] Yamashiro K Tanaka R Miyazaki S Miyauchi K Hayashi H Nishizaki Y. Comparison of primary and secondary stroke prevention in patients with nonvalvular atrial fibrillation: results from the raffine registry. J Stroke Cerebrovasc Dis (2022) 31(12):106871. doi: 10.1016/j.jstrokecerebrovasdis.2022.106871 36356431

[3] Lozano R Naghavi M Foreman K Lim S Shibuya K Aboyans V. Global and regional mortality from 235 causes of death for 20 age groups in 1990 and 2010: a systematic analysis for the global burden of disease study 2010. Lancet (2012) 380(9859):2095–128. doi: 10.1016/S0140-6736(12)61728-0 PMC10790329 23245604

[4] Krafft PR Bailey EL Lekic T Rolland WB Altay O Tang J. Etiology of stroke and choice of models. Int J Stroke (2012) 7(5):398–406. doi: 10.1111/j.1747-4949.2012.00838.x 22712741 PMC6986354

[5] Campbell B Khatri P. Stroke. Lancet (2020) 396(10244):129–42. doi: 10.1016/S0140-6736(20)31179-X 32653056

[6] Barthels D Das H. Current advances in ischemic stroke research and therapies. Biochim Biophys Acta Mol Basis Dis (2020) 1866(4):165260. doi: 10.1016/j.bbadis.2018.09.012 31699365 PMC6981280

[7] Miyamoto S Ogasawara K Kuroda S Itabashi R Toyoda K Itoh Y. Japan stroke society guideline 2021 for the treatment of stroke. Int J Stroke (2022) 17(9):1039–49. doi: 10.1177/17474930221090347 PMC9615334 35443847

[8] Kase CS Hanley DF. Intracerebral hemorrhage: advances in emergency care. Neurol Clin (2021) 39(2):405–18. doi: 10.1016/j.ncl.2021.02.002 33896526

[9] de Oliveira MA. Surgery for spontaneous intracerebral hemorrhage. Crit Care (2020) 24(1):45. doi: 10.1186/s13054-020-2749-2 32033578 PMC7006102

[10] Zufferey A Fontana P Reny JL Nolli S Sanchez JC. Platelet proteomics. Mass Spectrom Rev (2012) 31(2):331–51. doi: 10.1002/mas.20345 22009795

[11] Shaik NF Regan RF Naik UP. Platelets as drivers of ischemia/reperfusion injury after stroke. Blood Adv (2021) 5(5):1576–84. doi: 10.1182/bloodadvances.2020002888 PMC7948278 33687431

[12] Patil S Darcourt J Messina P Bozsak F Cognard C Doyle K. Characterising acute ischaemic stroke thrombi: insights from histology, imaging and emerging impedance-based technologies. Stroke Vasc Neurol (2022) 7(4):353–63. doi: 10.1136/svn-2021-001038 PMC9453827 35241632

[13] Njoroge FG Chen KX Shih NY Piwinski JJ. Challenges in modern drug discovery: a case study of boceprevir, an hcv protease inhibitor for the treatment of hepatitis c virus infection. Acc Chem Res (2008) 41(1):50–9. doi: 10.1021/ar700109k 18193821

[14] Macalino SJ Gosu V Hong S Choi S. Role of computer-aided drug design in modern drug discovery. Arch Pharm Res (2015) 38(9):1686–701. doi: 10.1007/s12272-015-0640-5 26208641

[15] Zhang Q Chen W Chen S Li S Wei D He W. Identification of key genes and upstream regulators in ischemic stroke. Brain Behav (2019) 9(7):e1319. doi: 10.1002/brb3.1319 PMC6625467 31168961

[16] Yang Z Wang G Luo N Tsang CK Huang L. Consensus clustering of gene expression profiles in peripheral blood of acute ischemic stroke patients. Front Neurol (2022) 13:937501. doi: 10.3389/fneur.2022.937501 35989931 PMC9388856

[17] Li Z Cui Y Feng J Guo Y. Identifying the pattern of immune related cells and genes in the peripheral blood of ischemic stroke. J Transl Med (2020) 18(1):296. doi: 10.1186/s12967-020-02463-0 32746852 PMC7398186

[18] Chen W Chen Y Wu L Gao Y Zhu H Li Y. Identification of cell death-related biomarkers and immune infiltration in ischemic stroke between male and female patients. Front Immunol (2023) 14:1164742. doi: 10.3389/fimmu.2023.1164742 37435058 PMC10332266

[19] Ma Z Liu CF Zhang L Xiang N Zhang Y Chu L. The construction and analysis of immune infiltration and competing endogenous rna network in acute ischemic stroke. Front Aging Neurosci (2022) 14:806200. doi: 10.3389/fnagi.2022.806200 35656537 PMC9152092

[20] Wang Y Cai Y. Obtaining human ischemic stroke gene expression biomarkers from animal models: a cross-species validation study. Sci Rep (2016) 6:29693. doi: 10.1038/srep29693 27407070 PMC4942769

[21] Zou R Zhang D Lv L Shi W Song Z Yi B. Bioinformatic gene analysis for potential biomarkers and therapeutic targets of atrial fibrillation-related stroke. J Transl Med (2019) 17(1):45. doi: 10.1186/s12967-019-1790-x 30760287 PMC6375208

[22] Li Q Wang R Yang Z Li W Yang J Wang Z. Molecular profiling of human non-small cell lung cancer by single-cell rna-seq. Genome Med (2022) 14(1):87. doi: 10.1186/s13073-022-01089-9 35962452 PMC9375433

[23] Liu Y Jiang H Kang T Shi X Liu X Li C. Platelets-related signature based diagnostic model in rheumatoid arthritis using wgcna and machine learning. Front Immunol (2023) 14:1204652. doi: 10.3389/fimmu.2023.1204652 37426641 PMC10327425

[24] Zhu W Shen Y Liu J Fei X Zhang Z Li M. Epigenetic alternations of micrornas and dna methylation contribute to gestational diabetes mellitus. J Cell Mol Med (2020) 24(23):13899–912. doi: 10.1111/jcmm.15984 PMC7753873 33085184

[25] Zhu T Wang L Tian F Zhao X Pu XP Sun GB. Anti-ischemia/reperfusion injury effects of notoginsenoside r1 on small molecule metabolism in rat brain after ischemic stroke as visualized by maldi-ms imaging. BioMed Pharmacother (2020) 129:110470. doi: 10.1016/j.biopha.2020.110470 32768957

[26] Xie W Zhu T Zhang S Sun X. Protective effects of gypenoside xvii against cerebral ischemia/reperfusion injury via sirt1-foxo3a- and hif1a-bnip3-mediated mitochondrial autophagy. J Transl Med (2022) 20(1):622. doi: 10.1186/s12967-022-03830-9 36572901 PMC9793669

[27] Yin Q Yang H Fang L Wu Q Gao S Wu Y. Fibroblast growth factor 23 regulates hypoxia−induced osteoblast apoptosis through the autophagy−signaling pathway. Mol Med Rep (2023) 28(5):199. doi: 10.3892/mmr.2023.13086 37711045 PMC10540001

[28] Yang Z Wei X Pan Y Xu J Si Y Min Z. A new risk factor indicator for papillary thyroid cancer based on immune infiltration. Cell Death Dis (2021) 12(1):51. doi: 10.1038/s41419-020-03294-z 33414407 PMC7791058

[29] Kawada JI Takeuchi S Imai H Okumura T Horiba K Suzuki T. Immune cell infiltration landscapes in pediatric acute myocarditis analyzed by cibersort. J Cardiol (2021) 77(2):174–8. doi: 10.1016/j.jjcc.2020.08.004 32891480

[30] Zheng PF Chen LZ Liu P Pan HW Fan WJ Liu ZY. Identification of immune-related key genes in the peripheral blood of ischaemic stroke patients using a weighted gene coexpression network analysis and machine learning. J Transl Med (2022) 20(1):361. doi: 10.1186/s12967-022-03562-w 35962388 PMC9373395

[31] Zhou H Qiu Z Gao S Chen Q Li S Tan W. Integrated analysis of expression profile based on differentially expressed genes in middle cerebral artery occlusion animal models. Int J Mol Sci (2016) 17(5):776. doi: 10.3390/ijms17050776 27213359 PMC4881595

[32] Zhang X Wang X Wang S Zhang Y Wang Z Yang Q. Machine learning algorithms assisted identification of post-stroke depression associated biological features. Front Neurosci (2023) 17:1146620. doi: 10.3389/fnins.2023.1146620 36968495 PMC10030717

[33] Wicik Z Czajka P Eyileten C Fitas A Wolska M Jakubik D. The role of mirnas in regulation of platelet activity and related diseases – a bioinformatic analysis. Platelets (2022) 33(7):1052–64. doi: 10.1080/09537104.2022.2042233 35285386

[34] Koupenova M Clancy L Corkrey HA Freedman JE. Circulating platelets as mediators of immunity. inflammation thrombosis Circ Res (2018) 122(2):337–51. doi: 10.1161/CIRCRESAHA.117.310795 PMC5777300 29348254

[35] Schlesinger M. Role of platelets and platelet receptors in cancer metastasis. J Hematol Oncol (2018) 11(1):125. doi: 10.1186/s13045-018-0669-2 30305116 PMC6180572

[36] Liu CD Liu NN Zhang S Ma GD Yang HG Kong LL. Salvianolic acid a prevented cerebrovascular endothelial injury caused by acute ischemic stroke through inhibiting the src signaling pathway. Acta Pharmacol Sin (2021) 42(3):370–81. doi: 10.1038/s41401-020-00568-2 PMC8027612 33303991

[37] Li Y Peng B Li Y Huang A Peng Y Yu Q. Mir-203a-3p/153-3p improves cognitive impairments induced by ischemia/reperfusion via blockade of src-mediated mapk signaling pathway in ischemic stroke. Chem Biol Interact (2022) 358:109900. doi: 10.1016/j.cbi.2022.109900 35305977

[38] George PM Mlynash M Adams CM Kuo CJ Albers GW Olivot JM. Novel tia biomarkers identified by mass spectrometry-based proteomics. Int J Stroke (2015) 10(8):1204–11. doi: 10.1111/ijs.12603 26307429

[39] Pruissen DM Slooter AJ Rosendaal FR van der Graaf Y Algra A. Coagulation factor xiii gene variation, oral contraceptives, and risk of ischemic stroke. Blood (2008) 111(3):1282–6. doi: 10.1182/blood-2007-08-110254 18006701

[40] Wang M Thomson AW Yu F Hazra R Junagade A Hu X. Regulatory t lymphocytes as a therapy for ischemic stroke. Semin Immunopathol (2023) 45(3):329–46. doi: 10.1007/s00281-022-00975-z PMC10239790 36469056

[41] Salas-Perdomo A Miro-Mur F Urra X Justicia C Gallizioli M Zhao Y. T cells prevent hemorrhagic transformation in ischemic stroke by p-selectin binding. Arterioscler Thromb Vasc Biol (2018) 38(8):1761–71. doi: 10.1161/ATVBAHA.118.311284 29903733

[42] Schuhmann MK Stoll G Bieber M Vögtle T Hofmann S Klaus V. Cd84 links t cell and platelet activity in cerebral thrombo-inflammation in acute stroke. Circ Res (2020) 127(8):1023–35. doi: 10.1161/CIRCRESAHA.120.316655 PMC7508294 32762491

[43] Schuhmann MK Guthmann J Stoll G Nieswandt B Kraft P Kleinschnitz C. Blocking of platelet glycoprotein receptor ib reduces "thrombo-inflammation" in mice with acute ischemic stroke. J Neuroinflamm (2017) 14(1):18. doi: 10.1186/s12974-017-0792-y PMC5251224 28109273

[44] Tyagi T Jain K Yarovinsky TO Chiorazzi M Du J Castro C. Platelet-derived tlt-1 promotes tumor progression by suppressing cd8+ t cells. J Exp Med (2023) 220(1):e20212218. doi: 10.1084/jem.20212218 36305874 PMC9814191

[45] Guo L Shen S Rowley JW Tolley ND Jia W Manne BK. Platelet mhc class i mediates cd8+ t-cell suppression during sepsis. Blood (2021) 138(5):401–16. doi: 10.1182/blood.2020008958 PMC8343546 33895821

[46] Li S Dou B Shu S Wei L Zhu S Ke Z. Suppressing nk cells by astragaloside iv protects against acute ischemic stroke in mice via inhibiting stat3. Front Pharmacol (2021) 12:802047. doi: 10.3389/fphar.2021.802047 35185544 PMC8852846

[47] Stivala S Reiner MF Lohmann C Luscher TF Matter CM Beer JH. Dietary alpha-linolenic acid increases the platelet count in apoe-/- mice by reducing clearance. Blood (2013) 122(6):1026–33. doi: 10.1182/blood-2013-02-484741 23801636

[48] Simpson IA Lorimer AR Walker ID Davidson JF. Effect of ciprofibrate on platelet aggregation and fibrinolysis in patients with hypercholesterolaemia. Thromb Haemost (1985) 54(2):442–4. doi: 10.1055/s-0038-1657868 4082082

[49] Gajdos M Mongiellova V Huttova D Cibulova L Krivosikova Z Spustova V. Ciprofibrate increases plasma concentration of platelet-derived growth factor ab in patients with advanced atherosclerosis and hyperlipidemia independently of its hypolipidemic effects. J Cardiovasc Pharmacol (2001) 38(5):651–6. doi: 10.1097/00005344-200111000-00001 11602811

