# Supplementary material for: Identification and validation of platelet-related diagnostic markers and potential drug screening in ischemic stroke by integrating comprehensive bioinformatics analysis and machine learning
Source: Front Immunol. 2024 Jan 10;14:1320475. doi: 10.3389/fimmu.2023.1320475 (PMC10806171; doi:10.3389/fimmu.2023.1320475)
Supplement: Supplementary file 2 [file DataSheet_2.zip › Supplementary Table 1.DOCX]

**Supplementary Table 1**. Sequences of primers for reverse transcription-quantitative PCR.

| Gene | Sequence, 5′à3′ | Base Counts |
| --- | --- | --- |
| App | FOR: CTGACCGAGGACTGACCACT | 20 |
|  | REV: TCATGTCCGAACTCCGCATC | 20 |
| Ppbp | FOR: TCCCGTGTGAATGTGTTCCG | 20 |
|  | REV: ATCATAGGGGCAGTCGGGT | 19 |
| Thbs1 | FOR: TTTGCGGAGAGGACACAGAC | 20 |
|  | REV: TCATAGTCTTCCTGCCCCGA | 20 |
| F13a1 | FOR: TGCAAATGGCTACAGCTCCA | 20 |
|  | REV: TGGACACCCTTTCCTTTGGG | 20 |
| Src | FOR: AGGACAGGTTGAGGCTGGTA | 20 |
|  | REV: AGGTGGGTAGAGTGGGTTGA | 20 |
| Vcl | FOR: ACCCTACAGTGGATGACCGA | 20 |
|  | REV: TCTACACGGTCACACTTGGC | 20 |
| Gapdh | FOR: AGAGACAGCCGCATCTTCTT | 20 |
|  | REV: TTCTCAGCCTTGACTGTGCC | 20 |
