# Supplementary material for: Identification and validation of platelet-related diagnostic markers and potential drug screening in ischemic stroke by integrating comprehensive bioinformatics analysis and machine learning
Source: Front Immunol. 2024 Jan 10;14:1320475. doi: 10.3389/fimmu.2023.1320475 (PMC10806171; doi:10.3389/fimmu.2023.1320475)
Supplement: Supplementary file 2 [file DataSheet_2.zip › Supplementary Table 2.DOCX]

**Supplementary Table 2.** GO and KEGG pathway enrichment of the lightgreen moudle.

| ONTOLOGY | ID | Description | p.adjust | Count |
| --- | --- | --- | --- | --- |
| BP | GO:0050878 | regulation of body fluid levels | 0.000013 | 10 |
| BP | GO:0042060 | wound healing | 0.000062 | 10 |
| BP | GO:0007596 | blood coagulation | 0.000005 | 9 |
| BP | GO:0050817 | coagulation | 0.000005 | 9 |
| BP | GO:0007599 | hemostasis | 0.000005 | 9 |
| BP | GO:0031589 | cell-substrate adhesion | 0.007248 | 7 |
| BP | GO:0007178 | transmembrane receptor protein serine/threonine kinase signaling pathway | 0.011851 | 7 |
| BP | GO:0030168 | platelet activation | 0.000386 | 6 |
| BP | GO:0007179 | transforming growth factor beta receptor signaling pathway | 0.005677 | 6 |
| BP | GO:0007160 | cell-matrix adhesion | 0.007248 | 6 |
| BP | GO:0071560 | cellular response to transforming growth factor beta stimulus | 0.011851 | 6 |
| BP | GO:0071559 | response to transforming growth factor beta | 0.012110 | 6 |
| BP | GO:0045860 | positive regulation of protein kinase activity | 0.022962 | 6 |
| BP | GO:0033674 | positive regulation of kinase activity | 0.031093 | 6 |
| BP | GO:0034329 | cell junction assembly | 0.032930 | 6 |
| BP | GO:0001894 | tissue homeostasis | 0.027947 | 5 |
| BP | GO:0060249 | anatomical structure homeostasis | 0.027947 | 5 |
| BP | GO:0090092 | regulation of transmembrane receptor protein serine/threonine kinase signaling pathway | 0.033004 | 5 |
| BP | GO:0036293 | response to decreased oxygen levels | 0.034944 | 5 |
| BP | GO:0007162 | negative regulation of cell adhesion | 0.036908 | 5 |
| BP | GO:0070482 | response to oxygen levels | 0.042115 | 5 |
| BP | GO:0048545 | response to steroid hormone | 0.043180 | 5 |
| BP | GO:0070527 | platelet aggregation | 0.007248 | 4 |
| BP | GO:0007044 | cell-substrate junction assembly | 0.012958 | 4 |
| BP | GO:0034109 | homotypic cell-cell adhesion | 0.012958 | 4 |
| BP | GO:0150115 | cell-substrate junction organization | 0.014949 | 4 |
| BP | GO:0007229 | integrin-mediated signaling pathway | 0.019181 | 4 |
| BP | GO:0045446 | endothelial cell differentiation | 0.021995 | 4 |
| BP | GO:0090100 | positive regulation of transmembrane receptor protein serine/threonine kinase signaling pathway | 0.022563 | 4 |
| BP | GO:0001952 | regulation of cell-matrix adhesion | 0.022563 | 4 |
| BP | GO:0001704 | formation of primary germ layer | 0.022962 | 4 |
| BP | GO:0003158 | endothelium development | 0.022962 | 4 |
| BP | GO:0050731 | positive regulation of peptidyl-tyrosine phosphorylation | 0.036549 | 4 |
| BP | GO:1901991 | negative regulation of mitotic cell cycle phase transition | 0.042115 | 4 |
| BP | GO:0007369 | gastrulation | 0.042115 | 4 |
| BP | GO:2000241 | regulation of reproductive process | 0.045052 | 4 |
| BP | GO:0045216 | cell-cell junction organization | 0.045052 | 4 |
| BP | GO:1901888 | regulation of cell junction assembly | 0.045052 | 4 |
| BP | GO:0009612 | response to mechanical stimulus | 0.045052 | 4 |
| BP | GO:0010810 | regulation of cell-substrate adhesion | 0.046445 | 4 |
| BP | GO:0032570 | response to progesterone | 0.014949 | 3 |
| BP | GO:0030195 | negative regulation of blood coagulation | 0.022563 | 3 |
| BP | GO:1900047 | negative regulation of hemostasis | 0.022563 | 3 |
| BP | GO:0050819 | negative regulation of coagulation | 0.022962 | 3 |
| BP | GO:0019755 | one-carbon compound transport | 0.022962 | 3 |
| BP | GO:0034332 | adherens junction organization | 0.022962 | 3 |
| BP | GO:0051932 | synaptic transmission, GABAergic | 0.022962 | 3 |
| BP | GO:0051893 | regulation of focal adhesion assembly | 0.027947 | 3 |
| BP | GO:0090109 | regulation of cell-substrate junction assembly | 0.027947 | 3 |
| BP | GO:0006081 | cellular aldehyde metabolic process | 0.031065 | 3 |
| BP | GO:0150116 | regulation of cell-substrate junction organization | 0.031065 | 3 |
| BP | GO:0030193 | regulation of blood coagulation | 0.031093 | 3 |
| BP | GO:1900046 | regulation of hemostasis | 0.031093 | 3 |
| BP | GO:0001707 | mesoderm formation | 0.032495 | 3 |
| BP | GO:0050818 | regulation of coagulation | 0.032495 | 3 |
| BP | GO:0048332 | mesoderm morphogenesis | 0.033004 | 3 |
| BP | GO:0043154 | negative regulation of cysteine-type endopeptidase activity involved in apoptotic process | 0.033004 | 3 |
| BP | GO:0061045 | negative regulation of wound healing | 0.033004 | 3 |
| BP | GO:0048041 | focal adhesion assembly | 0.039914 | 3 |
| BP | GO:2000134 | negative regulation of G1/S transition of mitotic cell cycle | 0.039914 | 3 |
| BP | GO:0033627 | cell adhesion mediated by integrin | 0.039914 | 3 |
| BP | GO:2000243 | positive regulation of reproductive process | 0.039914 | 3 |
| BP | GO:0097581 | lamellipodium organization | 0.042115 | 3 |
| BP | GO:2000117 | negative regulation of cysteine-type endopeptidase activity | 0.042115 | 3 |
| BP | GO:0051781 | positive regulation of cell division | 0.042822 | 3 |
| BP | GO:1903035 | negative regulation of response to wounding | 0.042822 | 3 |
| BP | GO:1902807 | negative regulation of cell cycle G1/S phase transition | 0.045052 | 3 |
| BP | GO:0048661 | positive regulation of smooth muscle cell proliferation | 0.045052 | 3 |
| BP | GO:2001237 | negative regulation of extrinsic apoptotic signaling pathway | 0.045052 | 3 |
| BP | GO:0032147 | activation of protein kinase activity | 0.049205 | 3 |
| BP | GO:0003348 | cardiac endothelial cell differentiation | 0.012110 | 2 |
| BP | GO:0060956 | endocardial cell differentiation | 0.012110 | 2 |
| BP | GO:0060717 | chorion development | 0.020656 | 2 |
| BP | GO:0003157 | endocardium development | 0.022962 | 2 |
| BP | GO:0010749 | regulation of nitric oxide mediated signal transduction | 0.022962 | 2 |
| BP | GO:1903867 | extraembryonic membrane development | 0.022962 | 2 |
| BP | GO:0046185 | aldehyde catabolic process | 0.027947 | 2 |
| BP | GO:0032230 | positive regulation of synaptic transmission, GABAergic | 0.029128 | 2 |
| BP | GO:0038166 | angiotensin-activated signaling pathway | 0.029128 | 2 |
| BP | GO:0090136 | epithelial cell-cell adhesion | 0.031065 | 2 |
| BP | GO:0010763 | positive regulation of fibroblast migration | 0.031093 | 2 |
| BP | GO:0051895 | negative regulation of focal adhesion assembly | 0.031093 | 2 |
| BP | GO:0150118 | negative regulation of cell-substrate junction organization | 0.031093 | 2 |
| BP | GO:0038128 | ERBB2 signaling pathway | 0.032495 | 2 |
| BP | GO:0045780 | positive regulation of bone resorption | 0.032495 | 2 |
| BP | GO:0032026 | response to magnesium ion | 0.033004 | 2 |
| BP | GO:0030220 | platelet formation | 0.039914 | 2 |
| BP | GO:0036120 | cellular response to platelet-derived growth factor stimulus | 0.042115 | 2 |
| BP | GO:0036344 | platelet morphogenesis | 0.042115 | 2 |
| BP | GO:0036119 | response to platelet-derived growth factor | 0.045052 | 2 |
| BP | GO:0042730 | fibrinolysis | 0.045052 | 2 |
| BP | GO:0007263 | nitric oxide mediated signal transduction | 0.045052 | 2 |
| BP | GO:1904385 | cellular response to angiotensin | 0.045052 | 2 |
| BP | GO:0010447 | response to acidic pH | 0.047288 | 2 |
| BP | GO:0032148 | activation of protein kinase B activity | 0.049539 | 2 |
| BP | GO:0001545 | primary ovarian follicle growth | 0.045052 | 1 |
| BP | GO:0006769 | nicotinamide metabolic process | 0.045052 | 1 |
| BP | GO:0060217 | hemangioblast cell differentiation | 0.045052 | 1 |
| BP | GO:0060957 | endocardial cell fate commitment | 0.045052 | 1 |
| BP | GO:0061445 | endocardial cushion cell fate commitment | 0.045052 | 1 |
| BP | GO:0150054 | regulation of postsynaptic neurotransmitter receptor diffusion trapping | 0.045052 | 1 |
| BP | GO:1900729 | regulation of adenylate cyclase-inhibiting opioid receptor signaling pathway | 0.045052 | 1 |
| BP | GO:1900731 | positive regulation of adenylate cyclase-inhibiting opioid receptor signaling pathway | 0.045052 | 1 |
| BP | GO:1901585 | regulation of acid-sensing ion channel activity | 0.045052 | 1 |
| BP | GO:1904702 | regulation of protein localization to adherens junction | 0.045052 | 1 |
| BP | GO:1990960 | basophil homeostasis | 0.045052 | 1 |
| BP | GO:2000476 | positive regulation of opioid receptor signaling pathway | 0.045052 | 1 |
| BP | GO:2001304 | lipoxin B4 metabolic process | 0.045052 | 1 |
| BP | GO:2001306 | lipoxin B4 biosynthetic process | 0.045052 | 1 |
| CC | GO:0031091 | platelet alpha granule | 0.000000 | 8 |
| CC | GO:0031093 | platelet alpha granule lumen | 0.000072 | 5 |
| CC | GO:0031092 | platelet alpha granule membrane | 0.000631 | 3 |
| CC | GO:0008305 | integrin complex | 0.003049 | 3 |
| CC | GO:0034774 | secretory granule lumen | 0.004897 | 6 |
| CC | GO:0060205 | cytoplasmic vesicle lumen | 0.004897 | 6 |
| CC | GO:0031983 | vesicle lumen | 0.004897 | 6 |
| CC | GO:0098636 | protein complex involved in cell adhesion | 0.009461 | 3 |
| CC | GO:0072562 | blood microparticle | 0.010248 | 4 |
| CC | GO:0005925 | focal adhesion | 0.012446 | 6 |
| CC | GO:0042581 | specific granule | 0.012446 | 4 |
| CC | GO:0030055 | cell-substrate junction | 0.012478 | 6 |
| CC | GO:0045177 | apical part of cell | 0.013581 | 6 |
| CC | GO:0030667 | secretory granule membrane | 0.017420 | 5 |
| CC | GO:0015629 | actin cytoskeleton | 0.018836 | 6 |
| CC | GO:0032587 | ruffle membrane | 0.024852 | 3 |
| CC | GO:0061826 | podosome ring | 0.026130 | 1 |
| CC | GO:0002102 | podosome | 0.026244 | 2 |
| CC | GO:0098978 | glutamatergic synapse | 0.036970 | 5 |
| CC | GO:0009897 | external side of plasma membrane | 0.036970 | 5 |
| CC | GO:0034684 | integrin alphav-beta5 complex | 0.036970 | 1 |
| CC | GO:0070443 | Mad-Max complex | 0.036970 | 1 |
| CC | GO:0071133 | alpha9-beta1 integrin-ADAM8 complex | 0.036970 | 1 |
| CC | GO:0071943 | Myc-Max complex | 0.036970 | 1 |
| CC | GO:0034683 | integrin alphav-beta3 complex | 0.047472 | 1 |
| CC | GO:0035866 | alphav-beta3 integrin-PKCalpha complex | 0.047472 | 1 |
| CC | GO:0035868 | alphav-beta3 integrin-HMGB1 complex | 0.047472 | 1 |
| CC | GO:0071062 | alphav-beta3 integrin-vitronectin complex | 0.047472 | 1 |
| MF | GO:0005178 | integrin binding | 0.000044 | 7 |
| MF | GO:0070051 | fibrinogen binding | 0.005840 | 2 |
| MF | GO:0070700 | BMP receptor binding | 0.024436 | 2 |
| MF | GO:0050840 | extracellular matrix binding | 0.024436 | 3 |
| MF | GO:0019838 | growth factor binding | 0.024436 | 4 |
| MF | GO:0070696 | transmembrane receptor protein serine/threonine kinase binding | 0.029012 | 2 |
| MF | GO:0030297 | transmembrane receptor protein tyrosine kinase activator activity | 0.032679 | 2 |
| MF | GO:0033612 | receptor serine/threonine kinase binding | 0.038537 | 2 |
| MF | GO:0017134 | fibroblast growth factor binding | 0.038537 | 2 |
| MF | GO:0050431 | transforming growth factor beta binding | 0.038537 | 2 |
| MF | GO:0016702 | oxidoreductase activity, acting on single donors with incorporation of molecular oxygen, incorporation of two atoms of oxygen | 0.038537 | 2 |
| MF | GO:0051213 | dioxygenase activity | 0.038537 | 3 |
| MF | GO:0016701 | oxidoreductase activity, acting on single donors with incorporation of molecular oxygen | 0.038537 | 2 |
| MF | GO:0004990 | oxytocin receptor activity | 0.038537 | 1 |
| MF | GO:0008112 | nicotinamide N-methyltransferase activity | 0.038537 | 1 |
| MF | GO:0018477 | benzaldehyde dehydrogenase (NADP+) activity | 0.038537 | 1 |
| MF | GO:0030760 | pyridine N-methyltransferase activity | 0.038537 | 1 |
| MF | GO:0038164 | thrombopoietin receptor activity | 0.038537 | 1 |
| MF | GO:0047977 | hepoxilin-epoxide hydrolase activity | 0.038537 | 1 |
| MF | GO:0001968 | fibronectin binding | 0.043180 | 2 |
| MF | GO:0030296 | protein tyrosine kinase activator activity | 0.049357 | 2 |
| KEGG | hsa04510 | Focal adhesion | 0.001399 | 7 |
| KEGG | hsa04512 | ECM-receptor interaction | 0.001399 | 5 |
| KEGG | hsa04611 | Platelet activation | 0.004578 | 5 |
| KEGG | hsa04350 | TGF-beta signaling pathway | 0.020258 | 4 |
| KEGG | hsa04613 | Neutrophil extracellular trap formation | 0.020258 | 5 |
| KEGG | hsa04810 | Regulation of actin cytoskeleton | 0.035336 | 5 |
| KEGG | hsa05418 | Fluid shear stress and atherosclerosis | 0.035336 | 4 |
| KEGG | hsa04151 | PI3K-Akt signaling pathway | 0.035499 | 6 |
| KEGG | hsa05412 | Arrhythmogenic right ventricular cardiomyopathy | 0.044356 | 3 |
| KEGG | hsa04610 | Complement and coagulation cascades | 0.054503 | 3 |
| KEGG | hsa05410 | Hypertrophic cardiomyopathy | 0.054834 | 3 |
| KEGG | hsa05222 | Small cell lung cancer | 0.054834 | 3 |
| KEGG | hsa05414 | Dilated cardiomyopathy | 0.056968 | 3 |
| KEGG | hsa05205 | Proteoglycans in cancer | 0.067915 | 4 |
| KEGG | hsa04015 | Rap1 signaling pathway | 0.067915 | 4 |
| KEGG | hsa05165 | Human papillomavirus infection | 0.067915 | 5 |
| KEGG | hsa04726 | Serotonergic synapse | 0.071509 | 3 |
| KEGG | hsa05219 | Bladder cancer | 0.073455 | 2 |
| KEGG | hsa04145 | Phagosome | 0.134567 | 3 |
| KEGG | hsa00590 | Arachidonic acid metabolism | 0.140400 | 2 |
| KEGG | hsa04060 | Cytokine-cytokine receptor interaction | 0.161402 | 4 |
| KEGG | hsa05100 | Bacterial invasion of epithelial cells | 0.193647 | 2 |
| KEGG | hsa01521 | EGFR tyrosine kinase inhibitor resistance | 0.193647 | 2 |
| KEGG | hsa04062 | Chemokine signaling pathway | 0.193647 | 3 |
| KEGG | hsa04012 | ErbB signaling pathway | 0.206525 | 2 |
| KEGG | hsa04727 | GABAergic synapse | 0.215698 | 2 |
| KEGG | hsa04520 | Adherens junction | 0.224693 | 2 |
| KEGG | hsa04750 | Inflammatory mediator regulation of TRP channels | 0.228793 | 2 |
| KEGG | hsa04640 | Hematopoietic cell lineage | 0.228793 | 2 |
| KEGG | hsa05163 | Human cytomegalovirus infection | 0.228793 | 3 |
| KEGG | hsa00360 | Phenylalanine metabolism | 0.230074 | 1 |
| KEGG | hsa00910 | Nitrogen metabolism | 0.236359 | 1 |
| KEGG | hsa04670 | Leukocyte transendothelial migration | 0.266931 | 2 |
| KEGG | hsa04919 | Thyroid hormone signaling pathway | 0.276989 | 2 |
| KEGG | hsa00340 | Histidine metabolism | 0.276989 | 1 |
| KEGG | hsa04964 | Proximal tubule bicarbonate reclamation | 0.280997 | 1 |
| KEGG | hsa04926 | Relaxin signaling pathway | 0.289804 | 2 |
| KEGG | hsa04966 | Collecting duct acid secretion | 0.310123 | 1 |
| KEGG | hsa01523 | Antifolate resistance | 0.333829 | 1 |
| KEGG | hsa00410 | beta-Alanine metabolism | 0.335692 | 1 |
| KEGG | hsa04921 | Oxytocin signaling pathway | 0.351268 | 2 |
| KEGG | hsa04110 | Cell cycle | 0.353868 | 2 |
| KEGG | hsa00350 | Tyrosine metabolism | 0.359204 | 1 |
| KEGG | hsa00760 | Nicotinate and nicotinamide metabolism | 0.360106 | 1 |
| KEGG | hsa04360 | Axon guidance | 0.418323 | 2 |
| KEGG | hsa00520 | Amino sugar and nucleotide sugar metabolism | 0.425386 | 1 |
| KEGG | hsa05202 | Transcriptional misregulation in cancer | 0.425386 | 2 |
| KEGG | hsa05167 | Kaposi sarcoma-associated herpesvirus infection | 0.425386 | 2 |
| KEGG | hsa05144 | Malaria | 0.425386 | 1 |
| KEGG | hsa04913 | Ovarian steroidogenesis | 0.425386 | 1 |
| KEGG | hsa05203 | Viral carcinogenesis | 0.440274 | 2 |
| KEGG | hsa04923 | Regulation of lipolysis in adipocytes | 0.457331 | 1 |
| KEGG | hsa04370 | VEGF signaling pathway | 0.457331 | 1 |
| KEGG | hsa04330 | Notch signaling pathway | 0.467068 | 1 |
| KEGG | hsa00310 | Lysine degradation | 0.467068 | 1 |
| KEGG | hsa00010 | Glycolysis / Gluconeogenesis | 0.468626 | 1 |
| KEGG | hsa05171 | Coronavirus disease - COVID-19 | 0.468626 | 2 |
| KEGG | hsa04917 | Prolactin signaling pathway | 0.468626 | 1 |
| KEGG | hsa05120 | Epithelial cell signaling in Helicobacter pylori infection | 0.468626 | 1 |
| KEGG | hsa00982 | Drug metabolism - cytochrome P450 | 0.468626 | 1 |
| KEGG | hsa04137 | Mitophagy - animal | 0.468626 | 1 |
| KEGG | hsa04115 | p53 signaling pathway | 0.468626 | 1 |
| KEGG | hsa05131 | Shigellosis | 0.468626 | 2 |
| KEGG | hsa04971 | Gastric acid secretion | 0.468626 | 1 |
| KEGG | hsa04020 | Calcium signaling pathway | 0.468626 | 2 |
| KEGG | hsa00980 | Metabolism of xenobiotics by cytochrome P450 | 0.468626 | 1 |
| KEGG | hsa03083 | Polycomb repressive complex | 0.491655 | 1 |
| KEGG | hsa04540 | Gap junction | 0.501180 | 1 |
| KEGG | hsa04976 | Bile secretion | 0.501180 | 1 |
| KEGG | hsa05032 | Morphine addiction | 0.503267 | 1 |
| KEGG | hsa04912 | GnRH signaling pathway | 0.505224 | 1 |
| KEGG | hsa04713 | Circadian entrainment | 0.509201 | 1 |
| KEGG | hsa01522 | Endocrine resistance | 0.509201 | 1 |
| KEGG | hsa04061 | Viral protein interaction with cytokine and cytokine receptor | 0.509201 | 1 |
| KEGG | hsa04972 | Pancreatic secretion | 0.509201 | 1 |
| KEGG | hsa05146 | Amoebiasis | 0.509201 | 1 |
| KEGG | hsa04625 | C-type lectin receptor signaling pathway | 0.510576 | 1 |
| KEGG | hsa05206 | MicroRNAs in cancer | 0.513039 | 2 |
| KEGG | hsa03013 | Nucleocytoplasmic transport | 0.513039 | 1 |
| KEGG | hsa04725 | Cholinergic synapse | 0.525289 | 1 |
| KEGG | hsa04724 | Glutamatergic synapse | 0.526076 | 1 |
| KEGG | hsa04660 | T cell receptor signaling pathway | 0.540875 | 1 |
| KEGG | hsa00230 | Purine metabolism | 0.551548 | 1 |
| KEGG | hsa04380 | Osteoclast differentiation | 0.551548 | 1 |
| KEGG | hsa04068 | FoxO signaling pathway | 0.551584 | 1 |
| KEGG | hsa04728 | Dopaminergic synapse | 0.551584 | 1 |
| KEGG | hsa04915 | Estrogen signaling pathway | 0.554276 | 1 |
| KEGG | hsa05135 | Yersinia infection | 0.554276 | 1 |
| KEGG | hsa04371 | Apelin signaling pathway | 0.554276 | 1 |
| KEGG | hsa04550 | Signaling pathways regulating pluripotency of stem cells | 0.559886 | 1 |
| KEGG | hsa04723 | Retrograde endocannabinoid signaling | 0.564647 | 1 |
| KEGG | hsa05226 | Gastric cancer | 0.564647 | 1 |
| KEGG | hsa04934 | Cushing syndrome | 0.564751 | 1 |
| KEGG | hsa04218 | Cellular senescence | 0.564751 | 1 |
| KEGG | hsa04390 | Hippo signaling pathway | 0.564751 | 1 |
| KEGG | hsa04514 | Cell adhesion molecules | 0.564751 | 1 |
| KEGG | hsa05161 | Hepatitis B | 0.569060 | 1 |
| KEGG | hsa04630 | JAK-STAT signaling pathway | 0.569752 | 1 |
| KEGG | hsa04022 | cGMP-PKG signaling pathway | 0.569752 | 1 |
| KEGG | hsa04530 | Tight junction | 0.571180 | 1 |
| KEGG | hsa05152 | Tuberculosis | 0.588447 | 1 |
| KEGG | hsa05034 | Alcoholism | 0.600198 | 1 |
| KEGG | hsa04814 | Motor proteins | 0.604939 | 1 |
| KEGG | hsa05130 | Pathogenic Escherichia coli infection | 0.609383 | 1 |
| KEGG | hsa05170 | Human immunodeficiency virus 1 infection | 0.624760 | 1 |
| KEGG | hsa05207 | Chemical carcinogenesis - receptor activation | 0.624760 | 1 |
| KEGG | hsa05417 | Lipid and atherosclerosis | 0.624760 | 1 |
| KEGG | hsa05166 | Human T-cell leukemia virus 1 infection | 0.625323 | 1 |
| KEGG | hsa05208 | Chemical carcinogenesis - reactive oxygen species | 0.625323 | 1 |
| KEGG | hsa04024 | cAMP signaling pathway | 0.625323 | 1 |
| KEGG | hsa04014 | Ras signaling pathway | 0.638090 | 1 |
| KEGG | hsa04144 | Endocytosis | 0.654474 | 1 |
| KEGG | hsa04010 | MAPK signaling pathway | 0.718795 | 1 |
| KEGG | hsa05014 | Amyotrophic lateral sclerosis | 0.776831 | 1 |
| KEGG | hsa04080 | Neuroactive ligand-receptor interaction | 0.776831 | 1 |
