# Supplementary material for: Identification and validation of platelet-related diagnostic markers and potential drug screening in ischemic stroke by integrating comprehensive bioinformatics analysis and machine learning
Source: Front Immunol. 2024 Jan 10;14:1320475. doi: 10.3389/fimmu.2023.1320475 (PMC10806171; doi:10.3389/fimmu.2023.1320475)
Supplement: Supplementary file 2 [file DataSheet_2.zip › Supplementary Table 3.DOCX]

**Supplementary Table 3.** List of PRGs in IS patients

| Number | Gene Symbol | Source | Expression |
| --- | --- | --- | --- |
| 1 | COL1A1 | Hub gene | Down-regulated |
| 2 | COL2A1 | Hub gene | Up-regulated |
| 3 | COL3A1 | Hub gene | Down-regulated |
| 4 | COL6A1 | Hub gene | Up-regulated |
| 5 | SPARC | Hub gene | Up-regulated |
| 6 | ITGB3 | Lightgreen module | Up-regulated |
| 7 | COL1A2 | Hub gene | Up-regulated |
| 8 | COL5A1 | Hub gene | Down-regulated |
| 9 | ALB | Hub gene | Up-regulated |
| 10 | A2M | Lightgreen module | Up-regulated |
| 11 | KNG1 | Cluster 2 | Up-regulated |
| 12 | APP | Cluster 2 | Up-regulated |
| 13 | PDGFRB | Hub gene | Up-regulated |
| 14 | PTGS1 | Lightgreen module | Up-regulated |
| 15 | ITGA2B | Lightgreen module | Up-regulated |
| 16 | PPBP | Lightgreen module | Up-regulated |
| 17 | PROS1 | Lightgreen module | Up-regulated |
| 18 | THBS1 | Lightgreen module | Up-regulated |
| 19 | F13A1 | Lightgreen module | Up-regulated |
| 20 | TAL1 | Lightgreen module | Up-regulated |
| 21 | ALOX12 | Lightgreen module | Up-regulated |
| 22 | MPL | Lightgreen module | Up-regulated |
| 23 | SRC | Lightgreen module | Up-regulated |
| 24 | VWF | Lightgreen module | Up-regulated |
| 25 | VCL | Lightgreen module | Up-regulated |
