# Supplementary material for: Identification and validation of platelet-related diagnostic markers and potential drug screening in ischemic stroke by integrating comprehensive bioinformatics analysis and machine learning
Source: Front Immunol. 2024 Jan 10;14:1320475. doi: 10.3389/fimmu.2023.1320475 (PMC10806171; doi:10.3389/fimmu.2023.1320475)
Supplement: Supplementary file 2 [file DataSheet_2.zip › Supplementary Table 4.DOCX]

**Supplementary Table 4.** Potential targeted therapeutic drugs for PRGs based on CMap analysis.

| Rank | Score | Type | ID | Name | Description |
| --- | --- | --- | --- | --- | --- |
| 8527 | -99.86 | cp | BRD-K33396764 | alpha-linolenic-acid | Omega 3 fatty acid stimulant |
| 8509 | -99.75 | cp | BRD-A49358627 | ciprofibrate | PPAR receptor agonist |
| 8501 | -99.72 | cp | BRD-K30189597 | SYK-inhibitor | SYK inhibitor |
| 8505 | -99.72 | cp | BRD-A09533288 | verapamil | Calcium channel blocker |
| 8491 | -99.66 | cp | BRD-K00184207 | GR-206 | Aryl hydrocarbon receptor ligand |
| 8487 | -99.61 | cp | BRD-K18895904 | olanzapine | Dopamine receptor antagonist |
| 8480 | -99.58 | cp | BRD-K49448285 | bisindolylmaleimide | CDK inhibitor |
| 8467 | -99.47 | cp | BRD-K84085265 | CG-930 | JNK inhibitor |
| 8460 | -99.4 | cp | BRD-K46862739 | metyrapone | Cytochrome P450 inhibitor |
| 8456 | -99.34 | cp | BRD-K89152108 | liothyronine | Thyroid hormone stimulant |
| 8454 | -99.33 | cp | BRD-K81376179 | TCS-359 | FLT3 inhibitor |
| 8443 | -99.23 | cp | BRD-K57926513 | tyrphostin-AG-1295 | PDGFR receptor inhibitor |
| 8442 | -99.22 | cp | BRD-K14643723 | 4-(2-Amino-ethyl)-benzenesulfonamide | carbonic anhydrase inhibitor |
| 8438 | -99.19 | cp | BRD-K40213712 | SAL-1 | Adenosine receptor antagonist |
| 8433 | -99.15 | cp | BRD-K22385716 | LY-303511 | Casein kinase inhibitor |
| 8430 | -99.12 | cp | BRD-K99451608 | lopinavir | HIV protease inhibitor |
| 8431 | -99.12 | cp | BRD-K63915849 | AS-604850 | PI3K inhibitor |
| 8426 | -99.08 | cp | BRD-K90382497 | GW-843682X | PLK inhibitor |
| 8425 | -99.07 | cp | BRD-K33483813 | actarit | Interleukin receptor agonist |
| 8415 | -98.97 | cp | BRD-A74771556 | nikkomycin | Chitin inhibitor |
| 8408 | -98.92 | cp | BRD-A53576514 | orphenadrine | Acetylcholine receptor antagonist |
| 8404 | -98.87 | cp | BRD-K15262564 | mupirocin | Isoleucyl-tRNA synthetase inhibitor |
| 8397 | -98.8 | cp | BRD-K05926469 | lenalidomide | Antineoplastic |
| 8394 | -98.79 | cp | BRD-K92731339 | perindopril | ACE inhibitor |
| 8390 | -98.73 | cp | BRD-K80527266 | triacsin-c | Adrenergic receptor antagonist |
| 8388 | -98.65 | cp | BRD-K97330509 | SRC-kinase-inhibitor-II | SRC inhibitor |
| 8387 | -98.63 | cp | BRD-K37312348 | kenpaullone | CDK inhibitor |
| 8376 | -98.52 | cp | BRD-K19416115 | sitagliptin | Dipeptidyl peptidase inhibitor |
| 8375 | -98.5 | cp | BRD-K53561341 | KIN001-220 | Aurora kinase inhibitor |
| 8374 | -98.48 | cp | BRD-K84266862 | BRL-50481 | Phosphodiesterase inhibitor |
