# Supplementary figures and images for: Identification and validation of platelet-related diagnostic markers and potential drug screening in ischemic stroke by integrating comprehensive bioinformatics analysis and machine learning
Source: Front Immunol. 2024 Jan 10;14:1320475. doi: 10.3389/fimmu.2023.1320475 (PMC10806171; doi:10.3389/fimmu.2023.1320475)

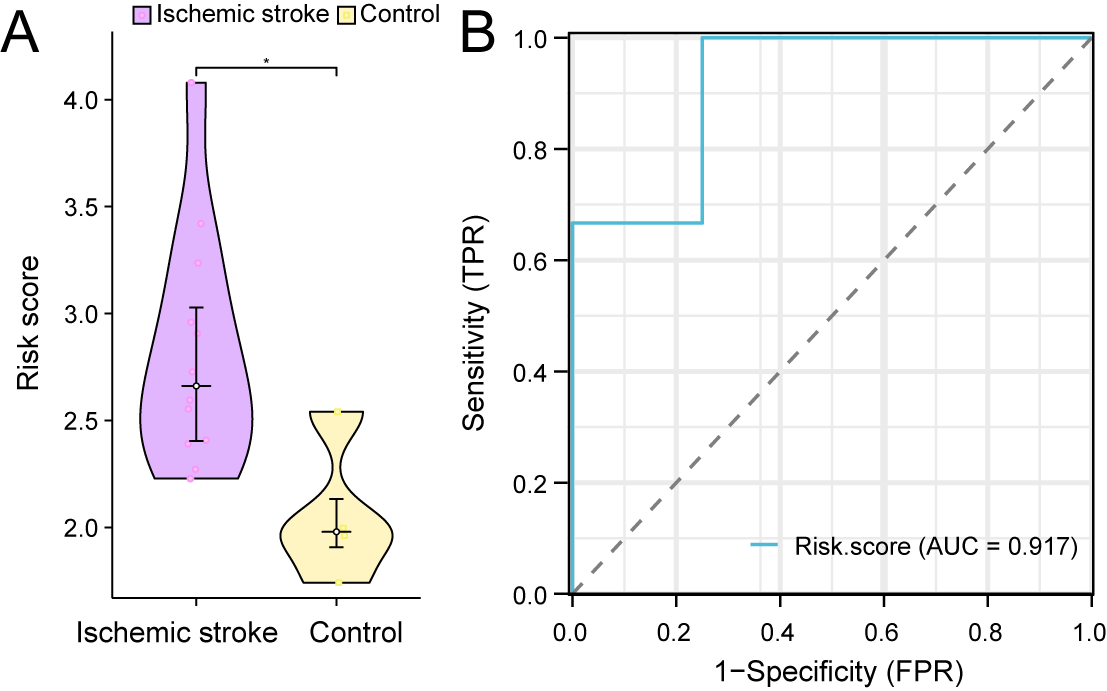

Supplement: Supplementary Figure 1 — Validation of platelet-related diagnostic models in GES202709. Risk scores of platelet-related diagnostic models for IS patients and healthy people in 202709 (A). ROC curve analysis of individual factors and diagnostic models of GES202709 (B). [file Image_1.tif]

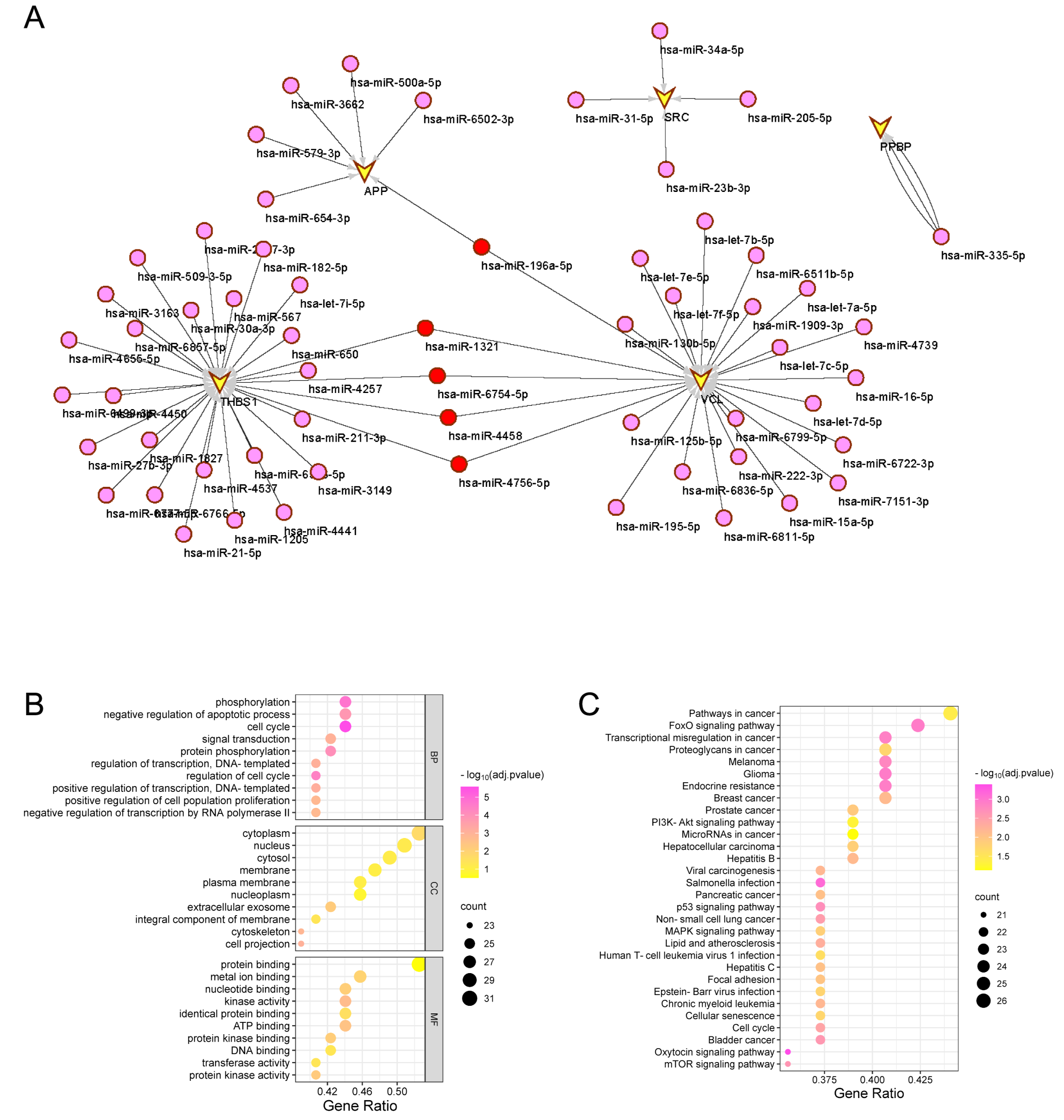

Supplement: Supplementary Figure 2 — Prediction and enrichment analysis of PADGS-associated miRNAs. Construction of the PADGS-related miRNA-mRNA network (A). The top 10 items in GOBP, CC, and MF (B) and the top 30 enriched pathways (C) in KEGG enrichment analysis of miRNAs. [file Image_2.tif]
